# Polyhydroxykanoate-Assisted Photocatalytic TiO_2_ Films for Hydrogen Production

**DOI:** 10.1021/acs.langmuir.4c02727

**Published:** 2024-11-22

**Authors:** Minoo Tasbihi, Sunil Kwon, Bumsoo Kim, Daniel Brüggemann, Heting Hou, Jiasheng Lu, Raffaele Amitrano, Thomas Grimm, Jordi García-Antón, Peter Strasser, Sebastian L. Riedel, Michael Schwarze

**Affiliations:** † Technische Universität Berlin, Department of Chemistry, Straße des 17, Juni 124, 10623 Berlin, Germany; ‡ Departament de Química, Unitat de Química Inorgànica, Universitat Autònoma de Barcelona, Bellaterra, 08193 Barcelona, Spain; § ANiMOX GmbH, Max-Planck-Straße 3, 12489 Berlin, Germany; ∥ Berliner Hochschule für Technik, Department VIII - Mechanical Engineering, Event Technology and Process Engineering, Environmental and Bioprocess Engineering Laboratory, 13353 Berlin, Germany

## Abstract

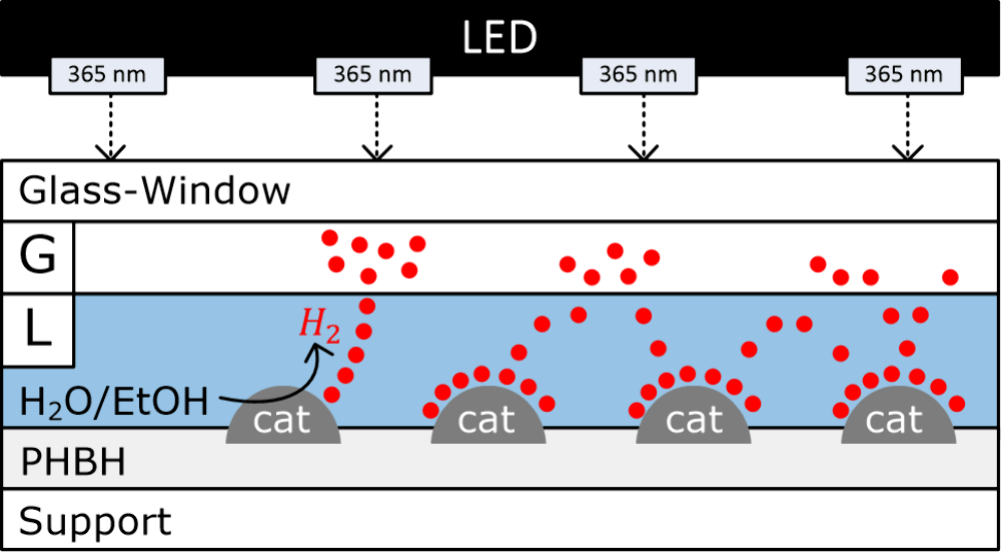

The photocatalytic production of hydrogen using biopolymer-immobilized titanium dioxide (TiO_2_) is an innovative and sustainable approach to renewable energy generation. TiO_2_, a well-known photocatalyst, benefits from immobilization on biopolymers due to its environmental friendliness, abundance, and biodegradability. In another way, to boost the efficiency of TiO_2_, its surface properties can be modified by incorporating co-catalysts like platinum (Pt) to improve charge separation. In this work, the surface of commercial TiO_2_ PC500 was modified with Pt nanoparticles (Pt1%@PC500) and then immobilized on glass surfaces using polyhydroxyalkanoate biopolymer poly(hydroxybutyrate-*co*-hydroxyhexanoate) (PHBH). The as-prepared immobilized Pt-modified TiO_2_ photocatalysts were fully characterized using various physicochemical techniques. The photocatalytic activity of the photocatalyst film was investigated for photocatalytic hydrogen production through water reduction using ethanol as a sacrificial donor. The impact of the film preparation conditions, e.g., PHBH concentration, PHBH:catalyst ratio, and temperature, on activity and stability was studied in detail. The application of biopolymer PHBH as a binder provides a green alternative to conventional immobilization methods, and with the application of PHBH, a stable and active photocatalyst film that showed lower activity compared to that of the suspended photocatalyst but good recyclability in six runs was prepared. A long-term photocatalytic hydrogen production experiment was carried out. In 98 h of operation, 12 mmol of hydrogen was produced in three consecutive runs with a PHBH/Pt1%@PC500 film having an area of ∼5.3 cm^2^. A significantly lower hydrogen productivity was observed after the first run, possibly due to a change in film structure, but thereafter, the productivity remained almost constant for the second and third runs. Hydrogen was the main product in the gas phase (90%), but carbon dioxide (4–5%) and methane (4–5%) were obtained as important byproducts. The byproducts are a consequence of the use of the sacrificial reagent ethanol. The results of the film performance are very promising, with regard to large-scale continuous hydrogen production.

## Introduction

The global demand for hydrogen (H_2_) gas has been steadily increasing due to its promising potential as a clean energy carrier using decarbonization in various sectors of the economy and enhancing the adoption of H_2_-based technologies. To meet this demand sustainably, there is a growing focus on expanding the production of low-carbon or green H_2_ produced from renewable sources and implementing policies and infrastructure to support its widespread use.^1,2^ Among various chemical engineering techniques and chemical industries such as steam-methane reforming and electrolysis for H_2_ production,^3^ the novelties and advances in the photocatalysis method have shown its promise. Photocatalytic H_2_ production is a process in which a photocatalyst is used to split water (H_2_O) molecules into H_2_ and oxygen (O_2_) under the influence of light. This is typically achieved using semiconducting materials as photocatalysts, which absorb light energy and promote the necessary chemical reactions. The general mechanism involves the absorption of photons by the photocatalyst material, which creates excited electron (e^–^)–hole (h^+^) pairs. These charge carriers then participate in redox reactions on the surface of the photocatalyst. Photocatalytic H_2_ production aims to develop efficient and cost-effective photocatalytic systems for sustainable hydrogen production, which holds promise as a clean and renewable energy source.^4,5^ To date, titania (TiO_2_) has been the most explored semiconductor for photocatalytic applications due to its outstanding chemical and thermal stability. However, it has both a large band gap energy and a relatively high rate of recombination of photoinduced (excited) e^–^–h^+^ pairs that lead to low photocatalytic activity. As a result, it would be significant to overcome these limitations and to improve the e^–^–h^+^ separation efficiency as well as the light utilization ability of TiO_2_.^4,5^ Surface modification of TiO_2_ with a noble metal is a common strategy employed by researchers.^4,5^ Noble metals like platinum (Pt) introduced as a co-catalyst onto the surface of titania act as electron scavengers and therefore inhibit e^–^–h^+^ recombination and improve the photocatalytic performance. In our previous research, Pt nanoparticles were introduced on titania particles by photodeposition,^6,7^ deposition and precipitation,^8^ and microemulsion.^9,10^ On the contrary, another important issue in the case of titania powders is the agglomeration of titania particles in photocatalytic reaction tests. An effective and practical strategy can be the immobilization and fixation of titania particles onto an immobilizing (supporting) material. The immobilization of titania powders enhances its stability and facilitates its reuse in photocatalytic reactions.^7,11−15^ However, the fabrication of nanoparticles without agglomeration, catalyst separation, and recovery in outstream is still under investigation. There are different immobilization methods such as the sol–gel method, microemulsion, and various substrates as immobilizing materials such as activated carbon,^16^ fiberglass cloth,^12^ an Al plate, silica gel, glass beads, silicate materials such as SBA-15,^17^ activated carbon,^16^ different zeolites,^18^ optical fibers,^19^ glass slides,^20^ aluminum sheets,^14^ glass beads,^21^ silica gel, and quartz sand.^21^ The choice of immobilization method depends on the specific application requirements, desired properties of the immobilized photocatalyst, and characteristics of the support material. Effective immobilization of TiO_2_ photocatalysts improves their performance, stability, and recyclability, making them suitable for various environmental and energy-related applications, such as water purification, air pollution control, and solar energy conversion. To transfer photocatalytic reactions from lab- to large-scale applications, the immobilization method should be easy, sustainable, and scalable. In addition, the obtained photocatalytic film should be active and stable. In this case, polymers can be used to immobilize catalyst materials that have the role of attaching the photocatalyst to the immobilizing material. Nafion is a well-known polymer used in catalyst immobilization^22^ and has been tested for immobilization of a carbon nitride photocatalyst for solar hydrogen production.^23^ To increase sustainability when polymers are used, biopolymers are a promising alternative.

Biopolymers from the polyhydroxyalkanoate (PHA) family are fully biodegradable in the environment, e.g., in soil or seawater, and therefore represent promising alternatives to conventional fossil-based polymers for the production of plastics.^24^ PHAs are linear polyesters that are stored by many microorganisms as carbon and energy reservoirs from a variety of carbonaceous raw materials.^25^ The properties of PHA are influenced by its molecular weight, which is usually between 0.1 × 10^6^ and 2 × 10^6^ kDa^26^ and monomer composition; for the latter, they are categorized in short-chain-length PHA (*scl*-PHA) with up to five carbon atoms and medium-chain-length (*mcl*-PHA) with six or more carbon atoms.^27^ The obtained monomer composition depends on the chosen production host and/or used carbon feedstock composition.^28^*scl*–*mcl* copolymer poly(3-hydroxybutyrate-*co*-3-hydroxyhexanoate) (PHBH) is of special interest for the production of foils or coatings, and its specific properties can be tuned by controlling the molar HHx content of the polymer.^29^ PHBH, which is produced from first-generation feedstocks, is commercially available from only very few companies, which is also because global commercial PHA production is only ∼100 kt/year at the moment.^30^ In contrast, ∼2 million tons of so-called bioplastics have been produced worldwide in 2023;^30^ however, this is still negligible compared to the global production of >400 million kt/year of petroleum-based plastics.^31^ To reduce the cost of production of PHA, the approaches focus on the use of biogenic waste streams, the optimization of production hosts through metabolic engineering, and the development of bioprocesses in monocultures and mixed cultures.^32,33^ Recently, we described PHBH production from animal-based side streams^34,35^ or plant-based renewable resources,^36^ which were used in this study as starting materials for photocatalytic TiO_2_ film production.

Herein, the commercial TiO_2_ modification PC500, decorated with platinum nanoparticles, was immobilized onto glass surfaces by using biopolymer PHBH. The obtained photocatalytic film was investigated for photocatalytic hydrogen production through water reduction using ethanol as a sacrificial donor. The impact of the film preparation conditions, e.g., PHBH concentration, PHBH:catalyst ratio, and temperature, on activity and stability was studied in detail to derive conditions for a stable and active photocatalytic film. The application of biomass-derived polymer PHBH together with the used photocatalytic systems is a green approach to H_2_ production.

## Experimental Section

### Materials

Poly(3-hydroxybutyrate-*co*-3-hydroxyhexanoate) with a molar HHx content of 14.9 mol % and an average molecular weight of 188 kDa, abbreviated as PHBH, was obtained from Animox GmbH (Berlin, Germany) (for production details, see the Supporting Information). Acetone (HPLC grade, VWR Chemicals) was used as the solvent to prepare the PHBH solutions. The TiO_2_ modification CrystalACTIV PC500 (Tronox France SAS) was used as the photocatalyst. Tris(dibenzylideneacetone)platinum(0) {[Pt(DBA)_3_], 98% pure, Strem Chemicals} was used as the precursor for platinum nanoparticles (PtNPs). Tetrahydrofuran (THF, 99.8% pure, Scharlab) was used as the solvent in co-catalyst deposition. H_2_ (>99% pure, Abelló Linde) was used as the reducing agent in co-catalyst deposition. Hexane (99% pure, Scharlab) was used as a cleaning solvent in co-catalyst deposition. Both solvents THF and hexane were first dried and distilled and then degassed via freeze–pump–thaw cycles before being used. Argon (5.0, Air Liquide) was used in photocatalytic H_2_ production to remove O_2_ before starting the irradiation process. Ethanol (EtOH, ≥99.8% pure, Roth) was used as the sacrificial agent in water splitting.

### Platinum Co-catalyst Deposition

An organometallic approach was employed to synthesize PtNPs on the surface of PC500. In detail, TiO_2_ was vacuum-dried at 80 °C to remove the adsorbed surface water and placed in a glovebox. Inside the glovebox, 396 mg of TiO_2_ was weighed together with 18.4 mg of [Pt(DBA)_3_] and placed in a 500 mL Fischer–Porter bottle. Then, 200 mL of THF was added. The reactor was pressurized with 3 bar of H_2_ and left to stir overnight at room temperature. Then, H_2_ was removed through a vacuum, and a carbon-coated copper grid (400 mesh) was prepared for transmission electron microscopy analysis by adding a single drop of the suspension. The samples were isolated by removing the THF solution with a cannula. Then, the solid was washed three times with hexane before being dried under vacuum. The prepared catalyst with a theoretical Pt loading of 1 wt % is abbreviated as Pt1%@PC500 hereafter.

### Film Preparation

**Figure 1:**
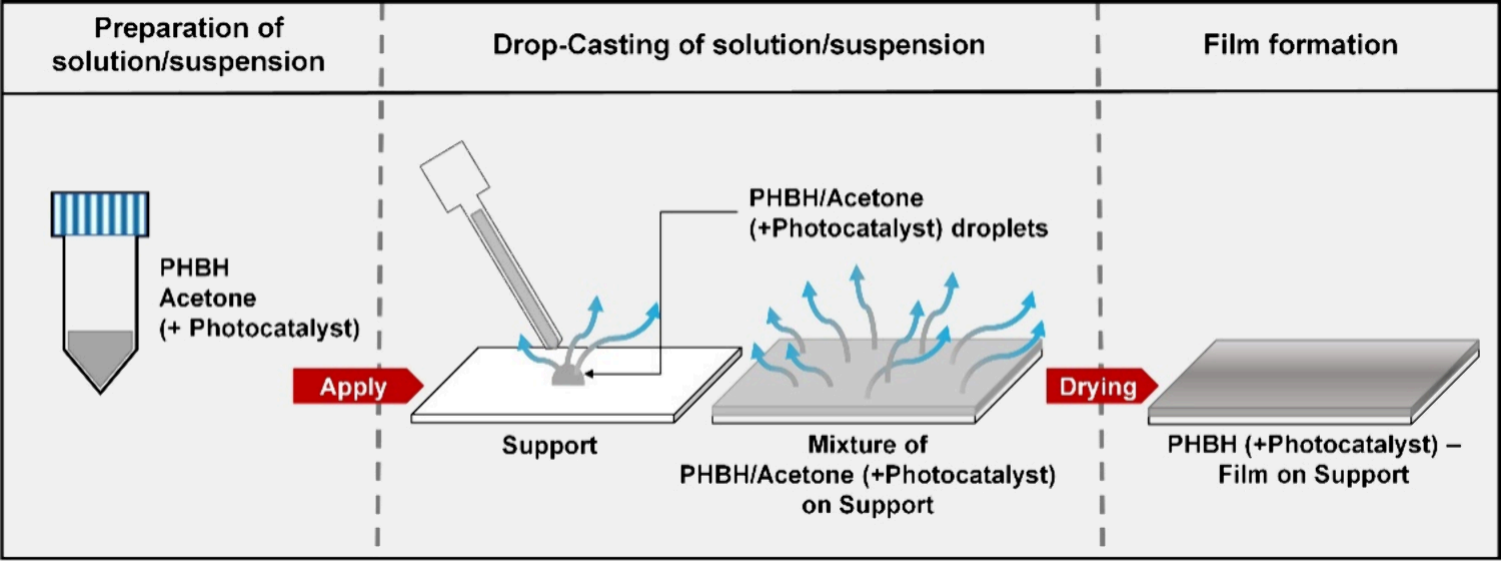
Scheme of film preparation using a drop coating procedure.

PHBH films without and with Pt1%@PC500 were obtained through drop coating. First, PHBH or PHBH and Pt1%@PC500 were mixed with acetone to obtain the desired concentrations. Second, the PHBH solution or the PHBH/Pt1%@PC500 suspension was drop coated onto a glass slide as the support material. If not mentioned otherwise, the dimensions of the glass slide were 2.6 cm × 3.6 cm, and the coated volume was 1 mL. In some cases, the solution or suspension was homogenized in an ultrasonic bath, drop coated at room temperature, and air-dried. In other cases, when the effect of temperature on film preparation was investigated, both the solution or suspension and the glass slide were set to the same temperature (Figure S1). The drop coating process is schematically shown in Figure 1.

### Photocatalytic Hydrogen Production

The photocatalytic Pt1%@PC500 films and the Pt1%@PC500 photocatalyst powder were investigated for H_2_ production in a top-irradiation reactor (TIR) that was connected to a thermostat (ministat 25, Huber) for temperature control. The experimental setup is shown in Figure S2. The photocatalyst powder or the photocatalytic film was placed in the TIR, and 20 mL of an aqueous solution containing 10 vol % EtOH was added. The reactor was closed with a quartz glass window, and the solution or suspension was purged for 15 min with argon to remove O_2_. The thermostat temperature was set to 20 °C, and a 365 nm ultraviolet (UV) light-emitting diode (LED) (400 W m^–2^, Neumüller Elektronik GmbH, Weisendorf, Germany), which was used as the artificial light source (lamp–reactor distance of 4 cm), was turned on. If not mentioned otherwise, irradiation was performed for 1 h. After the reaction, a sample of the gas phase was collected with a gastight syringe. The amount of H_2_ in the headspace after the photocatalytic experiment was measured by gas chromatography (GC) using an Agilent Technologies 7890 A instrument equipped with a Carboxen 1000 column and a thermal conductivity detector (TCD). The amount of H_2_ was calculated from eq 1.
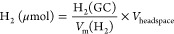
1where H_2_(GC) is the amount of H_2_ detected by GC, *V*_headspace_ is the headspace volume (105 mL), and *V*_m_(H_2_) is the molar volume of H_2_ (24.0 L mol^–1^ for 20 °C).

H_2_ production rates *r*_C_ (based on catalyst concentration) and *r*_A_ (based on irradiation area) were calculated from eqs 2 and 3, respectively.
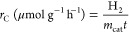
2
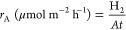
3where *m*_cat_ is the mass of Pt1%@PC500, *t* is the irradiation time, and *A* is the area irradiated by the UV LED.

### Analytical Methods

Thermogravimetric analysis (TGA) measurements were performed on a Mettler Toledo TGA/DSC 3+ instrument in 150 μL aluminum oxide crucibles. The temperature was varied from 25 to 300 °C using a heating rate of 5 K min^–1^. An isothermal section of 2 min at 300 °C was used at the end. Two separate measurements were carried out in the presence of either O_2_ or nitrogen (N_2_) at a gas flow rate of 20 mL min^–1^.

Differential scanning calorimetry (DSC) measurements were performed on a PerkinElmer Teller Pyris DSC-6 instrument in PerkinElmer Stainless Steel capsules with a capacity of 60 μL. Measurements were taken from 30 to 300 °C with a heating rate of 1 K min^–1^ under a nitrogen atmosphere (nitrogen flow rate of 60 mL min^–1^).

^1^H nuclear magnetic resonance (NMR) and ^13^C NMR spectra were recorded in a Norell 502 instrument on a Bruker Avance II 400 MHz spectrometer with a DUL 5 mm double-resonance probe (^1^H, ^13^C, Z-gradient, ATM). For sample preparation, a spatula tip of PHBH was mixed with 1 mL of acetone-*d*_6_.

Attenuated total reflectance-Fourier transform infrared (ATR-FTIR) spectroscopy was performed using a Bruker Vektor 22 ATR-IR instrument from 500 to 4000 cm^–1^ with a resolution of 1 cm^–1^.

Powder and thin film X-ray diffraction (XRD) measurements were conducted on Bruker D8 Advance instruments using Cu K radiation (λ = 1.5406 Å; LynxEye, Karlsruhe, Germany). The diffraction patterns were collected in the 2θ angle range of 10–70° (Bragg–Brentano geometry for the PXRD) with a step size of ∼0.04°. Reflections were assigned using the PDFMaintEX library (version 9.0.133).

The UV–visible (UV–vis) spectrum of PHBH was recorded with a Lambda 365 nm spectrometer from PerkinElmer.

The contact angle was measured with a setup from dataphysics GmbH (OCA15+ with LDU and Software SCA20).

The weight percent of Pt in Pt1%@PC500 was measured by inductively coupled plasma optical emission spectrometry (ICP-OES) using a PerkinElmer Optima 4300DV model system located in the Chemical Analyses Service (UAB), Spain.

## Results and Discussion

### Preparation of PHBH Films

**Figure 2:**
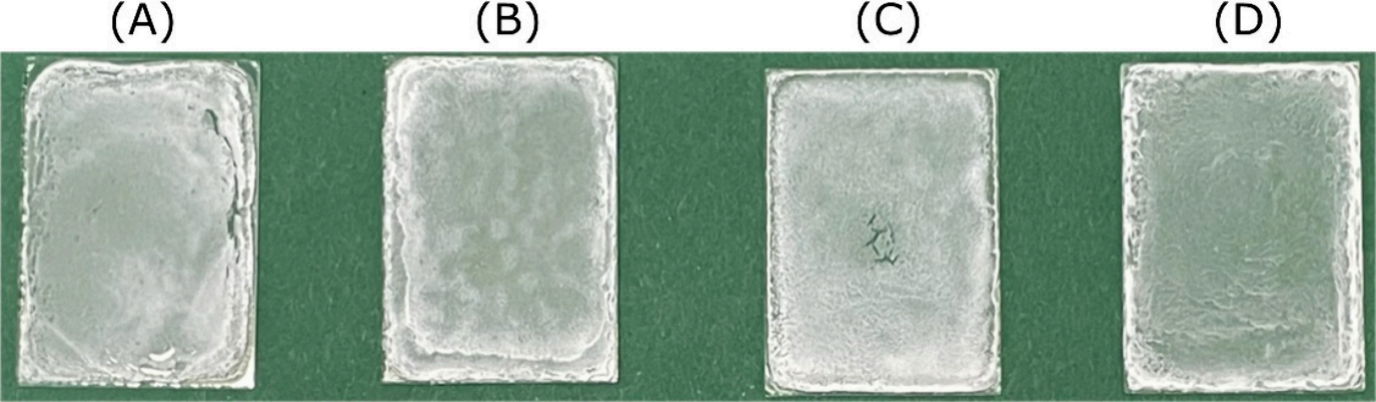
PHBH films on glass (*B* × *H* = 2.6 cm × 3.6 cm) prepared at room temperature (∼20 °C) at PHBH concentrations of (A) 10, (B) 2, (C) 1, and (D) 0.5 g L^–1^ (*m*_PHBH_ = 10 mg).

Biopolymer PHBH with a molar HHx content of 14.9 mol % and an average molecular weight of 188 kDa was obtained from Animox GmbH as a white fluffy material, whose optical appearance is strongly reminiscent of styrofoam (Figure S4). A brief description of PHBH with a base structural characterization, including ^1^H NMR, ^13^C NMR, UV–vis, and FTIR (Figure S5), and its temperature stability (Figure S6) are presented in section 2 of the Supporting Information. PHBH could be dissolved in acetone to prepare a casting solution. Before photocatalytic films with PHBH were prepared, the film-forming performance of PHBH was studied in the absence of the photocatalyst. Therefore, PHBH solutions were prepared by mixing the required amounts of PHBH and acetone, and after sonication, the PHBH solution was drop coated onto a glass slide at room temperature. The immobilized PHBH films are shown in Figure 2.

**Figure 3:**
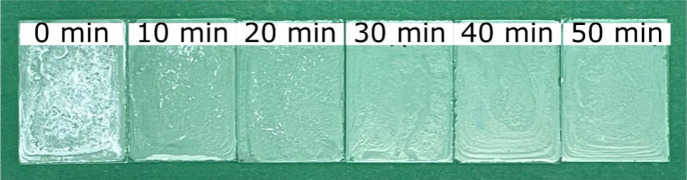
PHBH films on glass (*B* × *H* = 2.6 cm × 3.6 cm) prepared at room temperature (∼20 °C) using different sonication times (*m*_PHBH_ = 25 mg; *V*_acetone_ = 1 mL).

In all samples, the amount of PHBH was kept constant at 10 mg and the volume of acetone was varied between 1 and 20 mL to obtain the required PHBH concentrations. The films are not transparent, and there are slight differences in their homogeneity. The film thickness was measured to be ∼40 μm. The drying process, in which acetone is transferred from the liquid to gas phase, was performed at ∼20 °C, which is far below the boiling point of acetone (bp = 56 °C). It seems that a film prepared from lower PHBH concentrations (Figure 2) is more homogeneous, but it takes more time to remove the larger amount of acetone. In addition, it was noticed that the film quality sometimes changed when casting the same PHBH concentration. This was unexpected, but it was possible to identify the temperature as the main reason for the fluctuating film quality. The temperature of the PHBH solution increased (∼0.4 °C min^–1^) with the time used to homogenize it in the sonicator. Figure 3 shows the PHBH films prepared using the same amount of PHBH and a constant glass temperature of 20 °C, but the solution was treated at different times in the sonicator before being dropped onto the glass substrate.

**Figure 4:**
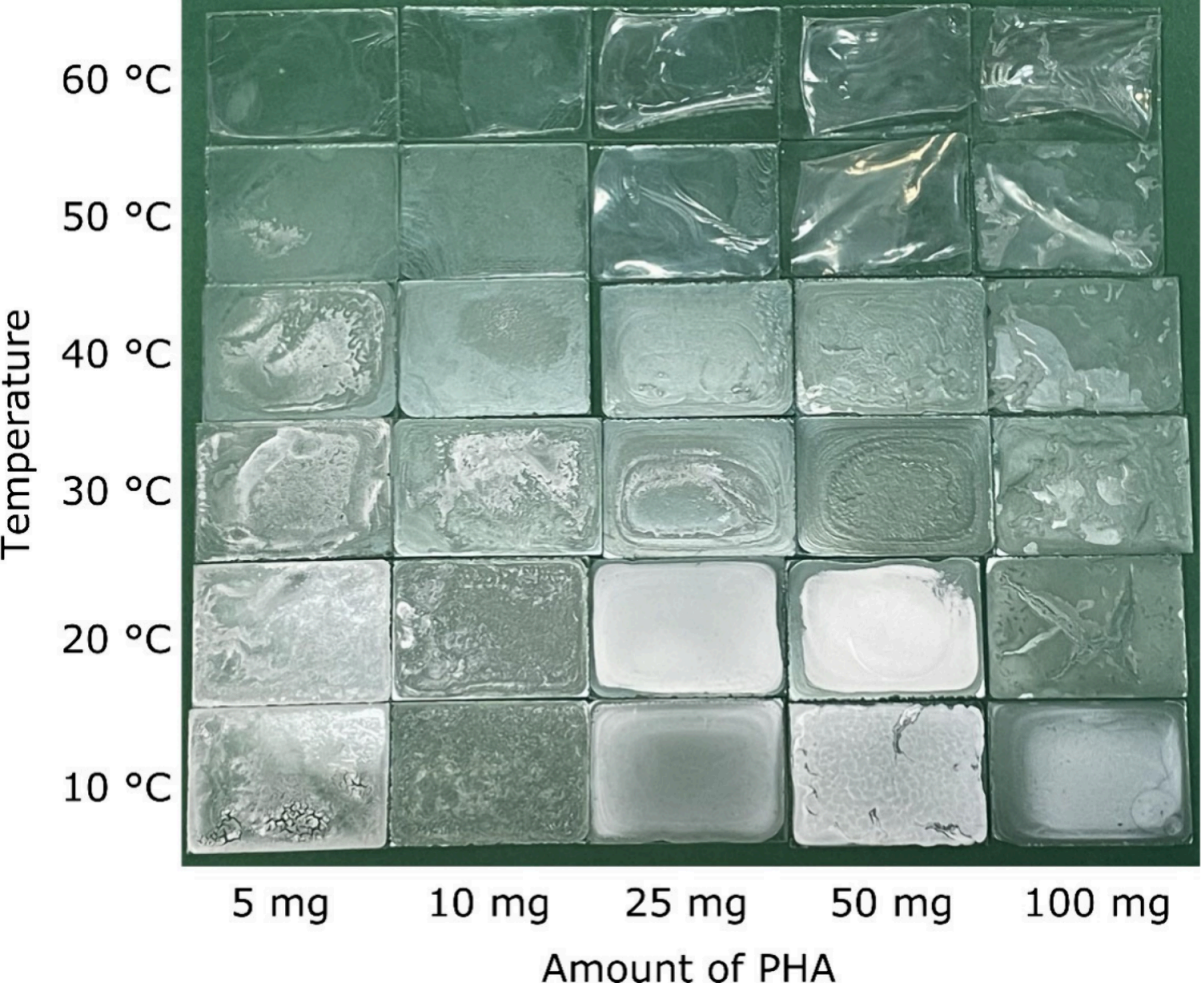
PHBH films on glass (*B* × *H* = 2.6 cm × 3.6 cm) were prepared at different PHBH concentrations and temperatures (*V*_acetone_ = 1 mL).

The film becomes more transparent and homogeneous with an increase in time in the sonicator, indicated by the removal of the white fragments shown for films prepared for sonicator times of 0–20 min. As the temperature was determined to have a huge effect on the film properties, PHBH films were prepared at defined concentrations and temperatures. In addition, the volume of acetone was kept constant at 1 mL which could be cast onto the glass surface in a single step (Figure 4).

The preparation conditions have a huge impact on film quality. It seems that there are three major trends. (i) The transparency increases with temperature. (ii) The film homogeneity increases with PHBH concentration. (iii) At higher PHBH concentrations and temperatures, the films detach from the glass surface, forming a PHBH foil. The transparency of PHBH films was measured by UV–vis spectroscopy, which confirmed the visual impression as shown in Figure S7. All prepared PHBH films have a hydrophobic surface as exemplarily shown in Figure S8, and the contact angle was determined to be ∼116°. Depending on the preparation conditions, the film thickness also changes. The film thickness increases with PHBH concentration, but for overly high PHBH concentrations, the film detaches from the surface as shown in Figure 4. The film thickness is exemplarily shown in Figure S10.

### 3.2 Preparation of PHBH/Pt1%@PC500 Films and Their Performance in Photocatalytic Hydrogen Production

**Figure 5:**
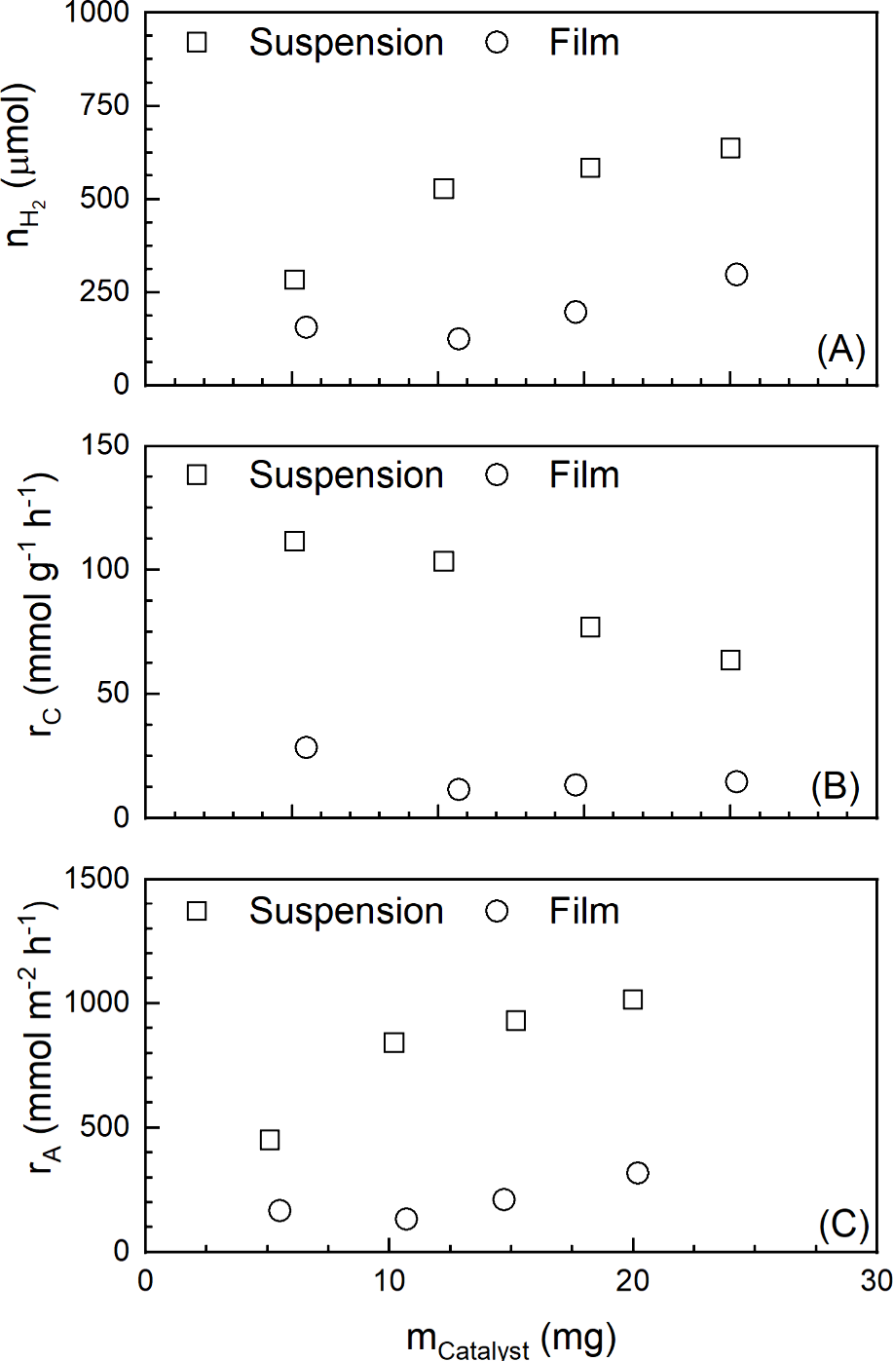
Photocatalytic hydrogen production with suspended Pt1%@PC500 particles and with PHBH/Pt1%@PC500 films (TIR; *V*_L_ = 20 mL; *V*_G_ = 105 mL; *t*_suspension_ = 30 min; *t*_film_ = 60 min; *T* = 20 °C; 365 nm UV LED; *m*_PHBH_ = 10 mg).

For photocatalytic hydrogen production, the TiO_2_ modification PC500 was modified with PtNPs as described in the Experimental Section (Pt1%@PC500). The mean Pt NPs particle size was ∼2.0 ± 0.4 nm, and the Pt NPs were homogeneously distributed over PC500 as shown in Figure S11. The nominal loading determined by ICP was 0.85 wt % Pt. In most cases, the TiO_2_ modification P25 that consists of rutile and anatase phases is investigated in photocatalytic experiments. In our previous investigation, we studied different commercial TiO_2_ modifications for photocatalytic hydrogen evolution, namely P25, P90, PC105, and PC500.^10^ Because of the much larger surface area of PC500, the Pt1%@PC500 photocatalyst showed a much higher rate of hydrogen production. Therefore, PC500 was selected in this study as the photocatalyst and decorated with platinum nanoparticles as the co-catalyst. To investigate the effect of PHBH as well as the impact of immobilization, experiments with suspended Pt1%@PC500 particles were carried out in the same photoreactor for comparison. The results for four different Pt1%@PC500 concentrations in the range of 0.25–1.0 g L^–1^ are shown in Figure 5.

As expected, the total amount of H_2_ increases with Pt1%@PC500 concentration because more light can be absorbed by the photocatalyst particles (Figure 5A). The rate of H_2_ production based on the mass of the photocatalyst (*r*_C_) was calculated, and *r*_C_ decreases with an increase in photocatalyst concentration (Figure 5B). This behavior is also expected because not all photocatalyst particles contribute equally to hydrogen production. When the photocatalyst concentration increases, a larger fraction of photocatalyst particles are in the shade and will not absorb light. In the case of reaction optimization, the photocatalyst concentration could be examined in terms of the point at which the absorption of light is optimized. Here, the optimal photocatalyst concentration seems to be ∼0.5 g L^–1^. However, the experiments with the suspended photocatalyst were performed only for comparison with the immobilized photocatalyst rather than to optimize the photocatalytic activity. When in the next step the Pt1%@PC500 photocatalysts are immobilized onto a glass slide as the support material, the particles cannot freely move in the reaction solution and *r*_C_ seems not to be the ideal value for comparison. It is better to relate the amount of H_2_ produced to the irradiated area (*r*_A_). As shown in Figure 5C, *r*_A_ has the same trend as the amount of hydrogen produced. Due to the top irradiation of the photocatalyst suspension, the particles close to the gas–liquid interphase will contribute more to H_2_ production than the deeper-lying ones. With an increase in photocatalyst concentration, the particle concentration at this interphase increases, leading to an increase in *r*_A_.

**Figure 6:**
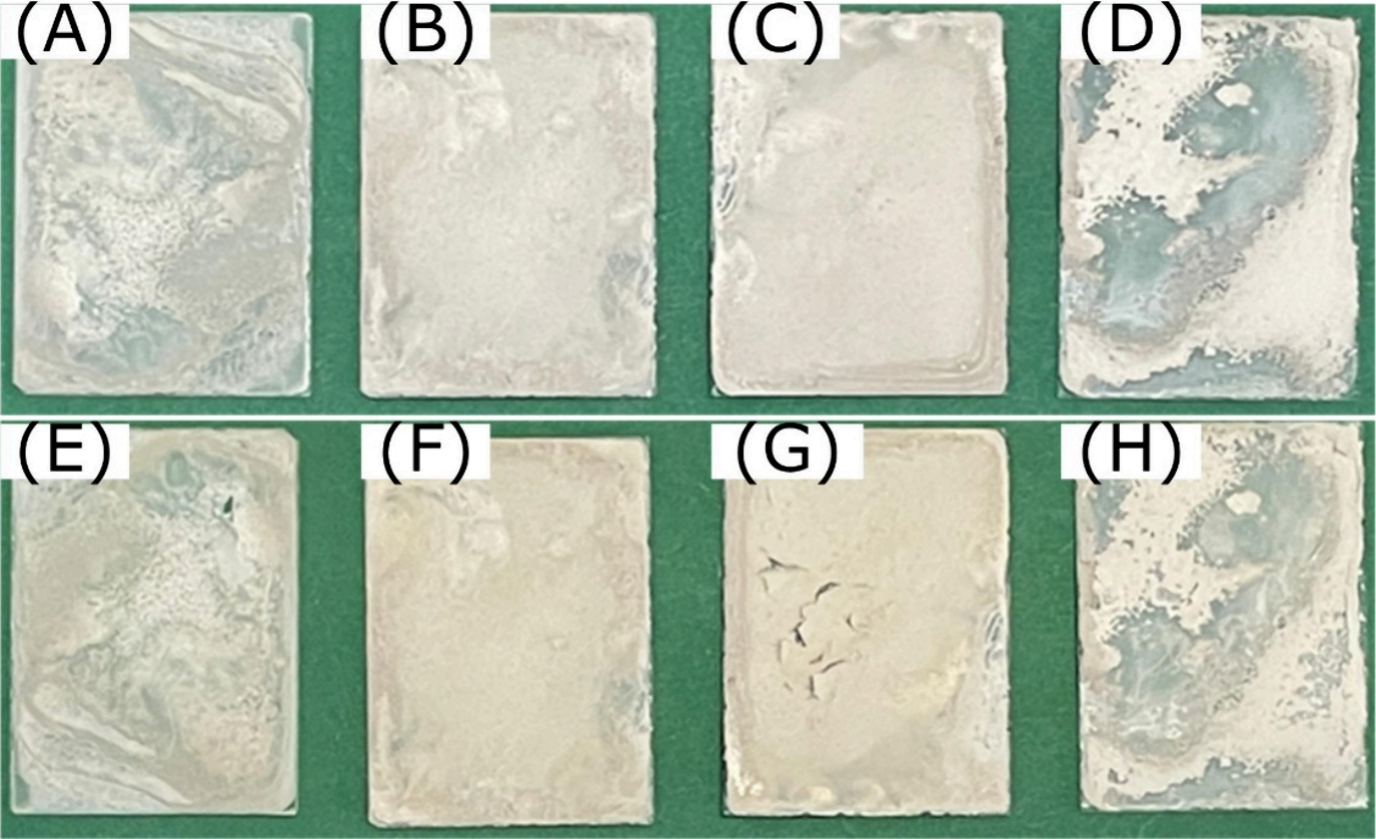
PHBH/Pt1%@PC500 films prepared on glass (*B* × *H* = 2.6 cm × 3.6 cm) (A–D) before and (E–H) after photocatalytic hydrogen production [*m*_Pt1%@PC500_ = (A and E) 5 mg, (B and F) 10 mg, (C and G) 15 mg, and (D and H) 20 mg; *m*_PHBH_ = 10 mg; *V*_Acetone_ = 1 mL; *T*_immobilization_ = 50 °C].

After the examination of the performance of Pt1%@PC500 particles, the same amount of Pt1%@PC500 particles (5–20 mg) was immobilized on a glass slide by using a fixed amount of PHBH as a polymeric binder (*m*_PHBH_ = 10 mg; *L*_PHBH_ = 1.1 mg cm^–2^). The immobilization was carried out at 50 °C, where the PHBH forms a transparent and homogeneous film, as shown in Figure 4. Figure 6 shows the visual behavior of the as-prepared samples before and after irradiation. The successful immobilization of the photocatalyst was verified by XRD measurements (Figure S9) showing diffraction peaks for PHBH (13.5° and 16.8°), the glass substrate (22°), and anatase TiO_2_ (25.0°, 37.9°, 47.7°, 54.2°, and 62.1°). The band gap energies (direct transition) for PC500, Pt1%@PC500, and PHBH/Pt1%@PC500 were also determined to check whether Pt as the co-catalyst or PHBH as the binder results in changes. As shown in Figure S12, the band gap energy is 3.1 eV in all cases. Thus, immobilization does not change the characteristic properties of the photocatalyst.

As Figure 6 makes obvious and as already discussed in section 3.2 of the Supporting Information for the PHBH films, the film quality strongly depends on the selected coating conditions. When the Pt1%@PC500 particles are added to the PHBH solution, the formation of a homogeneous film is more difficult. When the particle concentration is low, as in Figure 6A, the particles cannot cover the whole area of the glass slide. When the catalyst concentration is high, as in Figure 6D, the particles should be able to cover the whole glass slide, but due to the deposition temperature of 50 °C, no homogeneous film was obtained. For the intermediate particle concentration, as shown in panels B and C of Figure 6, the quality of the prepared films is good. Although the quality of the films is not perfect, all immobilized photocatalytic films are active with respect to hydrogen production (as exemplified in Figure S13), and the results are shown in Figure 5.

**Figure 7:**
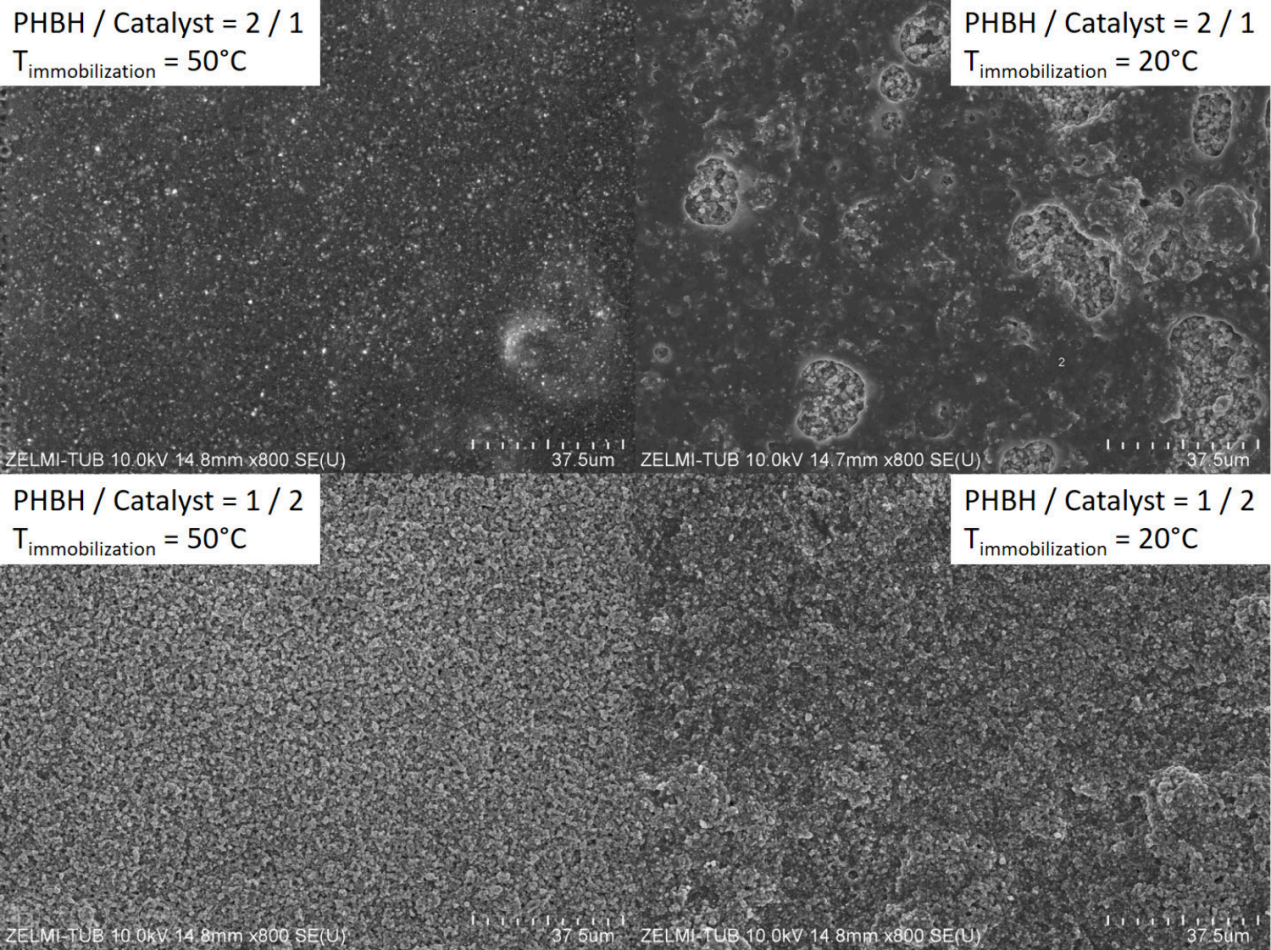
SEM images (top view) of PHBH/Pt1%@PC500 films on glass (2 cm × 2 cm) prepared at different PHBH:Pt1%@PC500 ratios (*m*_PHBH_ = 4.3 mg) and temperatures.

The trend for the films is similar to that observed for the suspension catalysts. The amount of H_2_ produced and *r*_A_ increase (Figure 5A,C), whereby *r*_C_ decreases (Figure 5B) with an increase in Pt1%@PC500 loading. When the amount of PHBH is kept constant and the Pt1%@PC500 loading is increased, more Pt1%@PC500 particles are accessible at the surface and can produce H_2_. Film formation was performed at 50 °C, which is not too far from the boiling point of acetone so that the solvent evaporates faster. Therefore, there is less time for the Pt1%@PC500 particles to sink into the formed PHBH film. However, at higher temperatures, the Pt1%@PC500 particles more strongly move, which might be the reason for the observed lack of homogeneity of the prepared films, especially at a higher catalyst loading. The decrease in *r*_C_ is due to the lower accessibility of the Pt1%@PC500 particles. Light absorption takes place in the upper layers of the prepared film, and particles immobilized in deeper layers do not contribute to H_2_ production. For these reasons, an immobilized catalyst layer should be (a) as thin as possible and (b) stable under operating conditions. Fulfilling both requirements for a catalytic film is not an easy task. Here, biopolymer PHBH was used as a binder to immobilize the Pt1%@PC500 particles on a glass slide surface. The amount of PHBH, the PHBH:Pt1%@PC500 ratio, and the deposition temperature are crucial parameters in the preparation of stable and active films. In the worst case, Pt1%@PC500 particles could be strongly covered by PHBH so that water has no access to them. To prove the effect of the PHBH:Pt1%@PC500 ratio, Pt1%@PC500 particles with the same PHBH:Pt1%@PC500 ratios as in Figure 6 were prepared at 20 and 50 °C. Figure S14 shows the optical behavior of the prepared films. The films prepared at 20 °C are more homogeneous because the evaporation of acetone is slow and particles are less mobile but can sink into the films, as is obvious from the white-colored areas at the film surface. For films prepared at 50 °C, the surface color is gray, indicating more Pt1%@PC500 particles are at the surface. Selected films, prepared at 20 and 50 °C, were characterized through SEM analysis as shown in Figure 7.

The SEM images clearly show that for films prepared from an excess of PHBH combined with a lower temperature, the surface is strongly covered by the polymer. This negatively affects the photocatalytic activity, as exemplified in Figure S15.

As mentioned above, the PHBH films are very hydrophobic, but the film becomes more hydrophilic after the addition of Pt1%@PC500 particles (Figure S8). The contact angle decreases from ∼116° to ∼80°, whereby it was observed that the contact angle decreases when the amount of Pt1%@PC500 is increased. A smaller contact angle compared to that of pure PHBH also indicates that the photocatalyst is present on the surface and accessible.

When the impact of PHBH on the activity of the prepared film was studied, it was found that in some cases the film was active but detached from the glass slide. This indicates that sometimes the PHBH film was not well fixed. It is necessary to have a good interaction between PHBH as a binder and the glass slide. Upon the addition of the photocatalyst particles, there will be interactions between the particles and between PHBH and the photocatalysts. Therefore, the main parameters that influence the stability, activity, and homogeneity of the film are the PHBH loading (*L*_PHBH_, milligrams per square centimeter), the PHBH:Pt1%@PC500 ratio, the PHBH concentration (*c*_PHBH_, milligrams per liter) of the casting solution, and the casting temperature (*T*, degrees Celsius). Also, the structure of the support surface (smooth, rough, etc.) can have an impact. The best performance was observed for a PHBH loading of ∼0.5 mg cm^–2^ (based on the 2.6 cm × 3.6 cm glass substrate), a PHBH:Pt1%@PC 500 ratio of 1:1–1:2, and a deposition temperature of 50 °C using a rough glass surface. A film was prepared using these conditions to study the long-term activity and stability. The PHBH/Pt1%@PC500 film was used in six individual runs, each having a duration of 3 h. The cumulative H_2_ production, the area-based H_2_ production, the observed mass losses, and the optical appearance of the film are shown in Figure S16. Constant H_2_ production with a mean of ∼0.5 mmol of H_2_ per run was obtained. The film quality was good, but weight loss was observed because some of the catalysts were not well fixed. The cumulative weight loss after six runs was ∼20%. As a consequence, the rate of H_2_ production decreased from 250 mmol h^–1^ m^–2^ (runs 1 and 2) to 180 mmol h^–1^ m^–2^ (runs 5 and 6). It has to be mentioned that the rate of H_2_ production is very high, and many gas bubbles are formed, which causes a greater stress on the film. Therefore, this result is very promising and it is assumed that the film performance might be further optimized through a detailed parameter study, including film-forming parameters and support materials.

**Figure 8:**
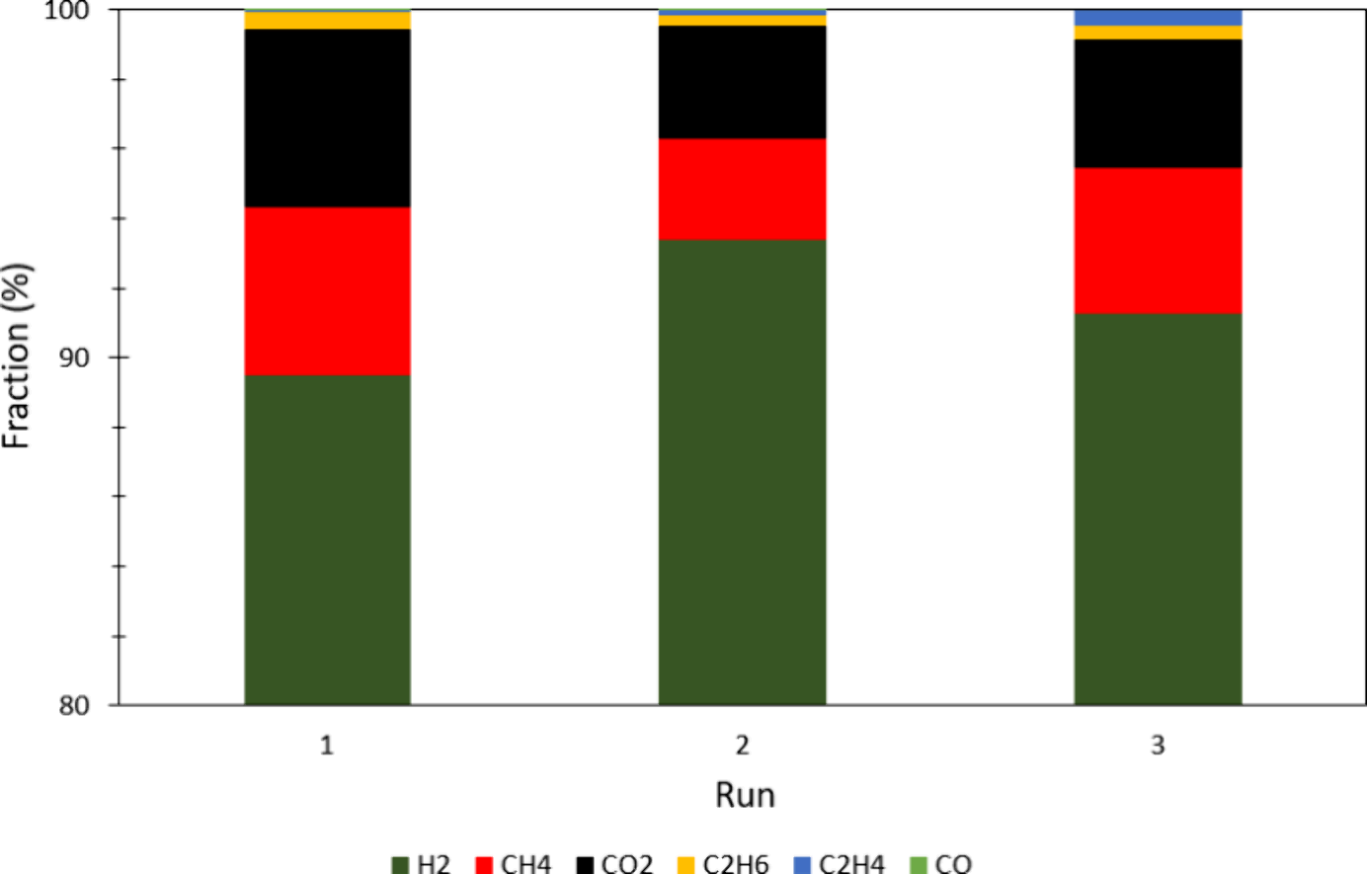
Composition of the gas phase after each run.

Because the first stability test with a total time of 18 h, divided into six runs of 3 h each, was short, a long-term test was carried out with the PHBH/Pt1%@PC500 film. The test was carried out with the PHBH/Pt15@PC500 film for 98 h, divided into three runs (24, 50, and 24 h). The photoreactor that was used could work with overpressures up to 1.5 bar and was capable of investigating film irradiation over longer periods of time under defined irradiation conditions (Figure S3). The film was prepared under optimized conditions by using a rough glass plate (1.5 cm × 3.5 cm) as the substrate, a PHBH loading of 0.5 mg cm^–2^, and a PHBH:Pt1%@PC500 ratio of 1:1. The ideal gas law was used to calculate the moles of produced gas from the obtained overpressure (see eq 1 in the Supporting Information and Figure S17). The largest amount of gas was produced during the first run (∼6 mmol over 24 h). In the second run, the amount of gas produced was significantly smaller (∼5 mmol in 50 h). The amount of gas produced in the third run was slightly smaller than that in the second run (∼2 mmol in 24 h). GC analysis after each run (Figure 8) revealed hydrogen as the main product (∼90%) and carbon dioxide (∼4–5%) and methane (∼4–5%) as the main byproducts. Smaller amounts of ethylene, ethane, and carbon monoxide were observed (Σ < 1%).

The amounts of individual gases (in millimoles) produced are listed in Table S2. As ethanol was used as the sacrificial agent in photocatalytic hydrogen production, C_2_H_4_ and C_2_H_6_ can be formed from its decomposition. The liquid phase was not analyzed in detail to check for further byproducts, but the pH of the solution after the first run was between 4 and 5, indicating the formation of an acidic byproduct, possibly acetic acid as previously mentioned in the literature.^37^ CO_2_ is a typical decomposition byproduct that can be due to the decomposition of the PHBH or ethanol. We assume that the CO_2_ is produced from ethanol because (a) ethanol is used in large quantities and can be more easily oxidized, (b) no mass loss of the film was observed after operation for 98 h, and (c) the CO_2_ content is directly related to H_2_ production. Methane is a byproduct that is obtained from the photocatalytic reaction between hydrogen and carbon dioxide.^38^

**Figure 9:**
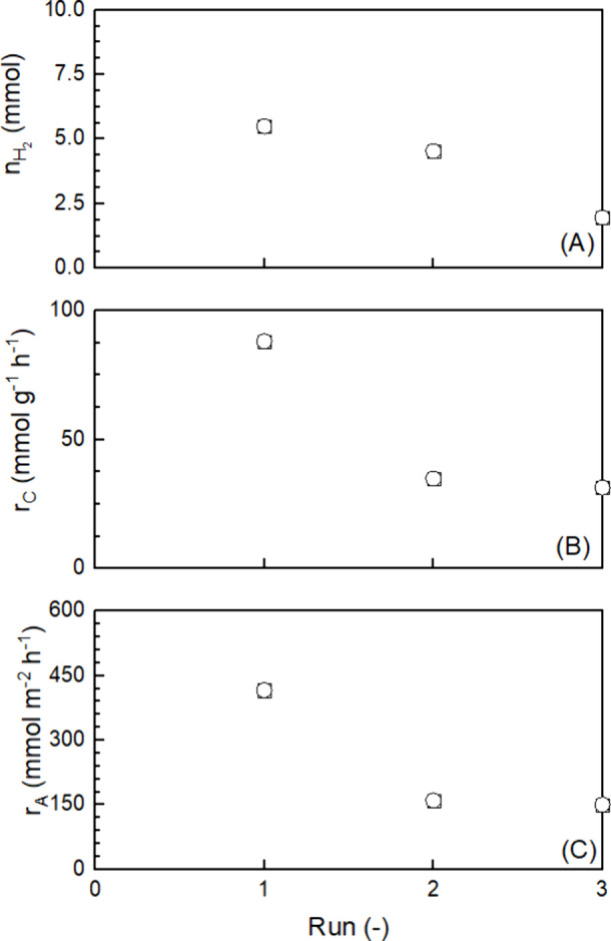
Long-term photocatalytic hydrogen production with a PHBH/Pt1%@PC500 film (*A* = 1.5 × 3.5 cm; *T* = 20 °C; 365 nm UV LED; *m*_Pt1%@PC500_ = *m*_PHBH_ = 2.6 mg; run 1 for 24 h; run 2 for 50 h; run 3 for 24 h).

The film consistently produced hydrogen. In total, ∼12 mmol of H_2_ was produced within 98 h (Figure 9A). The rate of hydrogen production for each run was calculated, and the results are shown in Figure 9. As already mentioned, the activity for the first run was high. The rate of hydrogen production was ∼90 mmol g^–1^ h^–1^ [related to the mass of the catalyst (Figure 9B)] or 400 mmol m^–2^ h^–1^ [related to the irradiated area (Figure 9C)]. The values are on the same order of magnitude, even better than those shown in Figure 5, because of a smaller amount of PHBH used to prepare the film. In the second run, the rates fell to approximately 40–50% of the original value but remained almost constant in the third run. The loss of activity is not attributed to a loss of material during the photocatalytic experiment as the weight of the samples was not changed. It is assumed that the structure of the film and thereby the location of the photocatalyst particles within the film have changed during the experiment. There are two possible reasons that need to be investigated further in the future. The first reason is related to the handling of the sample between the individual runs. To restart the experiment, the reactor was evacuated and filled with argon up to three times. During this treatment, when the film is strongly wetted from the experiment, the photocatalyst/PHBH distribution might have changed. This reason seems to make sense, as the activity remained constant during a run, even if it lasted 50 h. The second reason could have to do with the irradiation of the film. Because of the side irradiation of the film, gas evolution is tangential to the sample and could entrain particles within the film. The film appeared darker at the edges and partially detached from the glass substrate. This effect could also be enhanced by the vacuum between the runs. Photos of the film before and after irradiation are shown in Figure S18.

Due to the promising results, a PHBH/Pt1%@PC500 film measuring 30 cm × 30 cm was immobilized on a stainless steel plate by means of spray coating, and hydrogen production was tested with sunlight. On sunny and warm days with a high UV index, noticeable hydrogen production is also visible. The reactor does not currently allow any quantification. A reconstruction of the photoreactor is planned, and further tests will be carried out seasonally in the coming summer.

The focus of this study was to investigate biopolymer PHBH as a binder to prepare photocatalytic films. With Pt1%@PC500, a photocatalyst was selected that has a very high activity for hydrogen production when ethanol is used as the sacrificial agent. As shown above, the use of PHBH does not change the properties of the photocatalyst, so films of other photocatalysts can be prepared and investigated. In the future, a photocatalyst that can split water in the absence of a sacrificial agent and possibly with visible light should be used. Most of the interesting photocatalysts are not commercially available; therefore, this study was conducted with the well-known TiO_2_ photocatalyst. Due to our experience with carbon nitride (CN) photocatalysts, we can discuss pros and cons for both systems. CN and TiO_2_ are stable photocatalysts. In our earlier contribution, ∼20 L of hydrogen was produced in one month in a large-scale photoreactor (∼1 m^2^) under sunlight irradiation.^23^ Carbon nitrides belong to a class of metal-free photocatalysts that are composed of carbon and nitrogen only. The band gap energy is ∼2.7 eV, meaning that it can absorb visible light. It requires an amine as a sacrificial agent for photocatalytic hydrogen production, which makes the reaction less sustainable. The standard sacrificial agent is triethanolamine (TEOA), and the reaction mixture is strongly basic.^23^ TiO_2_ will not show hydrogen production with visible light as the band gap energy is ∼3.1 eV, but it can operate with ethanol, which makes the whole photocatalytic process more sustainable as ethanol can be obtained from renewables. The long-term lab-scale experiment using the PHBH/Pt1%@PC500 film produced 12 mmol of hydrogen (area of 5.25 cm^2^), which is ∼0.3 L. The suspended photocalyst shows even higher activity but is in general difficult to recycle. It is expected that on a sunny and warm day with a higher UV index, the TiO_2_/ethanol system outperforms carbon nitride/TEOA and can produce ≤500 L in 2 weeks (1 m^2^ area). The most important challenge is to produce a stable and active photocatalyst film. Spray coating is a well-known technology, and as mentioned above, a spray-coated PHBH/Pt1%@PC500 film showed already qualitative gas evolution with sunlight.

## Conclusions

The 1 wt % Pt-modified TiO_2_ (Pt1%@PC500) photocatalysts have been successfully immobilized on glass using PHBH as a biopolymer material. The mean Pt NP particle size was ∼2.0 ± 0.4 nm, and the Pt NPs were homogeneously distributed over titania. The actual amount of Pt NPs determined by ICP was 0.85 wt % Pt. To find the optimum stable and photocatalytically effective immobilized photocatalyst, the amount of PHBH and preparation (sonication) temperature were first varied by 5–100 mg and 10–60 °C, respectively. The Pt1%@PC500 photocatalyst powder and the glass-immobilized Pt1%@PC500 films were investigated for H_2_ production. In the suspension system, an optimum photocatalyst concentration of 0.5 mg L^–1^ was obtained, and immobilized Pt1%@PC500 showed photocatalytic activity behavior similar to that of the suspension photocatalyst. The amount of PHBH, the PHBH:Pt1%@PC500 ratio, and the deposition temperature are crucial parameters in the preparation of stable and active films. Thus, a PHBH loading of ∼0.5 mg cm^–2^, a PHBH:Pt1%@PC 500 ratio of 1:1–1:2, and a deposition temperature of 50 °C using a rough glass surface were shown to be the optimum conditions. The long-term activity and stability of an optimum film were also investigated in detail, showing a good recyclability of the photocatalyst film in six consecutive runs. As the rate of H_2_ production is high, the major challenge is to remove the H_2_ from the film as quickly as possible without the need for a long path through the polymer, which can lead to detachment of the photocatalytic film, especially if the polymer is not bound sufficiently strongly to the surface. A long-term experiment with a PHBH/Pt1%@PC500 film successfully produced 12 mmol of hydrogen in three runs with a total irradiation time of 98 h. While there was a clear difference in activity between the first and second runs, the performance for the third run remained almost constant. Possible causes could be the handling between the runs or strong H_2_ formation within the reaction, which led to a change in the film properties. Even if hydrogen is the main product of the reaction, decomposition and secondary products (e.g., CO_2_ and CH_4_) could be identified in the long-term experiment due to ethanol as the sacrificial reagent. A more favorable composition of the gas phase should result, if a photocatalyst is used that can split water without the help of a sacrificial reagent, e.g., strontium titanate (SrTiO_3_). The results are promising, and large-scale experiments with real sunlight irradiation are planned for the future.

## Supporting Information Available

Experimental setups, production and characterization (UV–vis, FTIR, ^1^H NMR, ^13^C NMR, and TGA) of PHBH, characterization (UV–vis absorption, contact angle, and XRD) of photocatalytic films, characterization of Pt1%@PC500, TEM/SEM description, and activity and stability of photocatalytic films (PDF)
