# Supplementary material for: Polyhydroxykanoate-Assisted Photocatalytic TiO2 Films for Hydrogen Production
Source: Langmuir. 2024 Nov 22;40(49):25800–10. doi: 10.1021/acs.langmuir.4c02727 (PMC11636239; doi:10.1021/acs.langmuir.4c02727)
Supplement: Supplementary file 1 — la4c02727_si_001.pdf [file la4c02727_si_001.pdf]

Polyhydroxyalkanoate -assisted photocatalytic TiO_2 films for hydrogen production

Supporting Information

Minoo Tasbihi[1], Sunil Kwon[1], Bumsoo Kim[1], Daniel Brüggemann[1], Heting Hou[2], Jiasheng Lu[1], Raffaele Amitrano[1], Thomas Grimm[3], Jordi García-Antón[2], Peter Strasser[1], Sebastian L. Riedel[4], Michael Schwarze[1]*

[1]Technische Universität Berlin, Department of Chemistry, Straße des 17. Juni 124, 10623 Berlin, Germany

[2]Departament de Química, Unitat de Química Inorgànica, Universitat Autònoma de Barcelona, 08193 Bellaterra, Barcelona, Spain

[3]ANiMOX GmbH, Max-Planck-Straße 3, 12489 Berlin, Germany

[4]Berliner Hochschule für Technik, Department VIII – Mechanical Engineering, Event Technology and Process Engineering, Environmental and Bioprocess Engineering Laboratory, 13353 Berlin, Germany

*corresponding author: Michael Schwarze (ms@chem.tu-berlin.de)

1. EXPERIMENTAL SETUPS

The setup to prepare the biopolymer or biopolymer/photocatalyst films at constant temperature is shown in **Figure S1**. The samples were prepared in 15 mL PET tubes (B) and placed into the reservoir (A). The glass substrate (D) was placed onto the bottom of a double-walled glass reactor. The reservoir and the double-walled reactor were connected in series with a thermostat, which was set to the requested temperature. 1 mL of the liquid sample was taken out of the PET tubes, dropped onto the glass substrate, and the glass substrate was removed after complete evaporation of the solvent acetone. It has to be mentioned that handling of the liquid samples at higher temperatures (boiling point acetone = 56 °C) is difficult because of fast acetone evaporation.

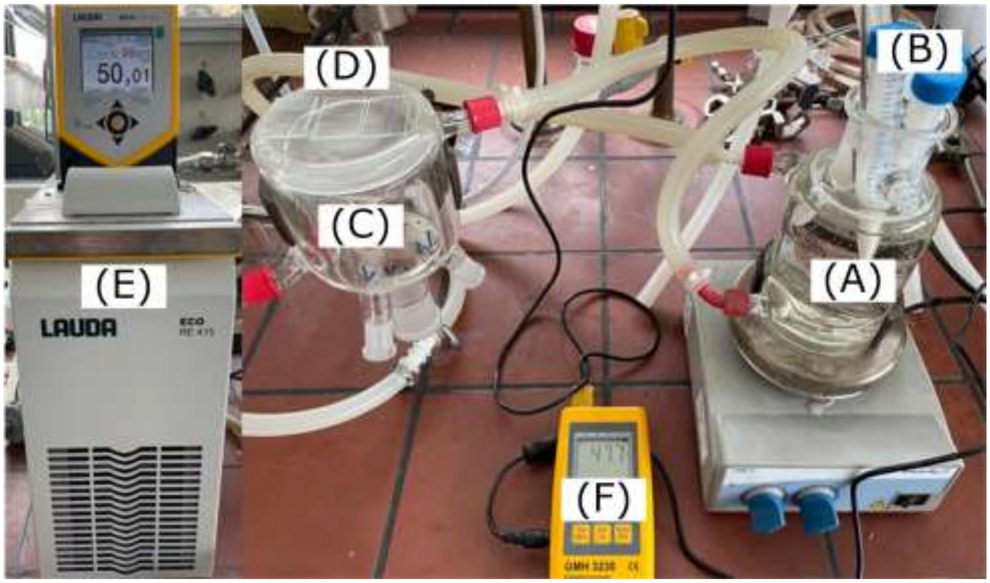

Figure S1. Setup for film preparation at constant temperature consisting of a reservoir (A) for tempering sample solutions (B), surface (C) for tempering the glass substrate (D), thermostat (E), and thermocouple (F).

The setup for photocatalytic hydrogen production with photocatalyst powders and photocatalyst films is shown in **Figure S2**. The photocatalyst as powder or film was placed into the top-irradiation reactor (1) and the aqueous solution containing ethanol was added. The solution was

purged with argon through a Schlenk-line (7) to remove oxygen. In the case of photocatalyst powders, the suspension was intensively stirred (4). The reaction temperature was set with a thermostat (5). The LED was turned on and photocatalyst was irradiated for a certain time (mostly 1h). The LED was turned off, a sample of the gas phase was taken with a gas-tight syringe through a septum (6), and the sample was analyzed by gas chromatography.

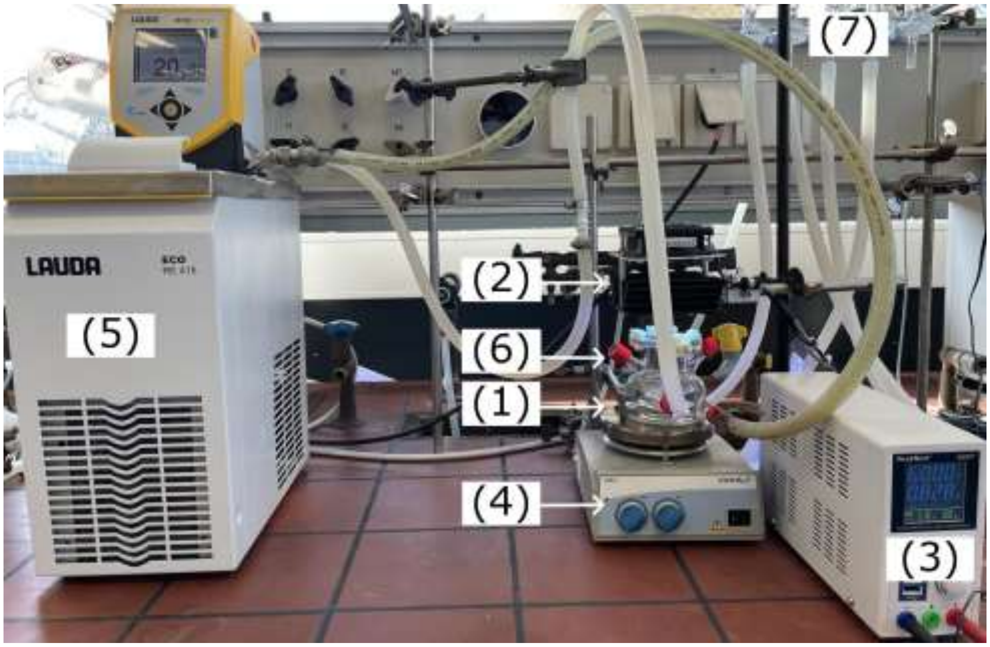

Figure S2. Setup for photocatalytic hydrogen production consisting of the top-irradiation photoreactor (1), a 365 nm UV-LED (2), the LED power supply (3), a stirrer for powder catalysts (4), a thermostat (5), sampling position (6), and a Schlenk-line (7) connected to an argon bottle.

The setup for investigating long-term photocatalytic hydrogen production is shown in **Figure S3**. It consists of a stainless-steel photoreactor (1) equipped with a Teflon inlet for the photocatalyst. Both suspended photocatalysts and photocatalyst films (6) can be examined. The sample can be irradiated with different light sources, e.g., with a 365 nm UV-LED (2). The temperature of the liquid phase during irradiation is controlled with a thermostat (4) and measured in the photoreactor with a thermocouple (7). For high gas production, a gas tank (150 mL) can be used (3) so that the total volume of the gas phase is approx. 165 mL. The pressure during irradiation is measured by a

pressure sensor (8). Pressure and temperature are recorded during the measurement. After the experiment, a sample of the gas phase can be taken for GC analysis (5).

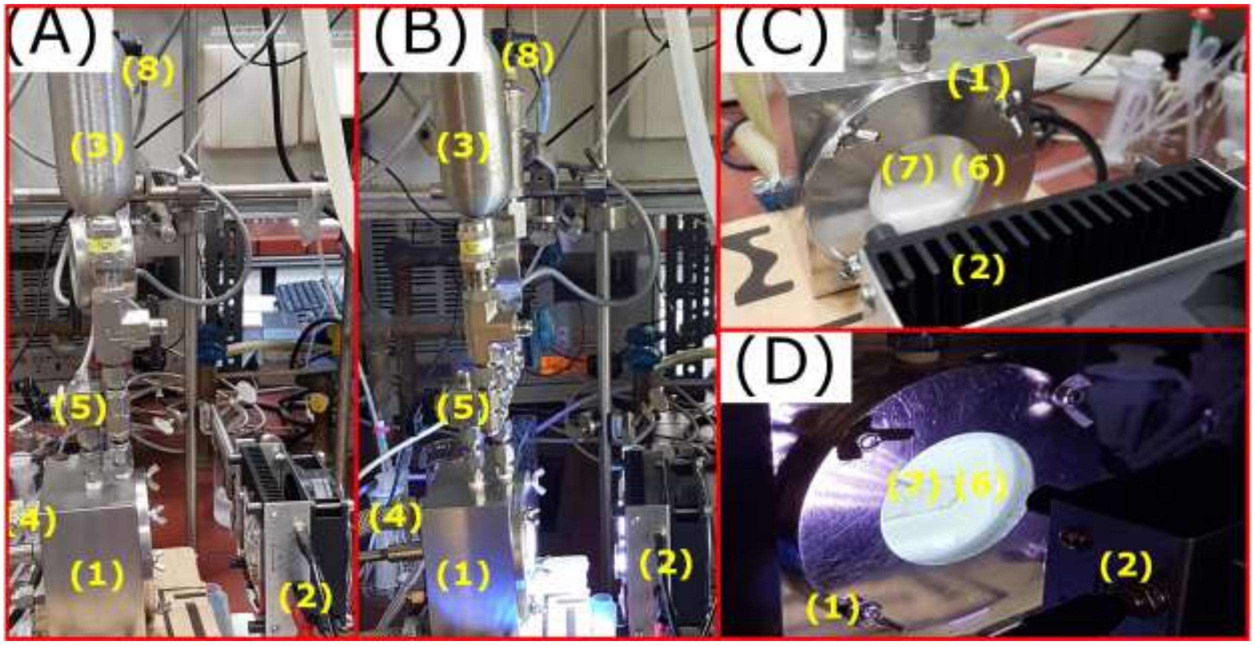

Figure S3. Setup for long-term photocatalytic hydrogen production before irradiation (A, C) and during irradiation (B, D). 1: photoreactor, 2: UV-LED, 3: gas reservoir (150 mL), 4: connection to thermostat, 5: sampling valve, 6: front view of reaction chamber with PHBH/Pt1%@PC500 film, 7: thermocouple, and 8: pressure sensor.

The photocatalytic film was placed inside of the photoreactor and the reactor was vacuumed through a Schlenk line (not shown) and filled with argon (3x). Approx. 38 mL of the aqueous solution containing ethanol was added under argon flow through the sampling valve. All valves (to the Schlenk line and sampling position) were closed and the LED lamp was switched on. The PC program was started to record pressure and temperature during the experiment. Three runs were performed and after each run a) a sample of the gas phase was taken for analysis, b) the liquid was removed from the reactor, and c) the reactor was refilled with fresh solution and restarted.

The moles of the generated gas were calculated from the recorded overpressure ($\Delta p(t)$) in the photoreactor according to Equation (1).

$$n_{gas}(t) = \frac{\Delta p(t) \cdot V_{gas}}{R \cdot T} \qquad (1)$$

In Equation (1), V_{gas} is the volume of the gas phase (approx. 165 mL), R is the ideal gas constant (8.314 J mol^{-1} K^{-1}), and T is the temperature (293.15 K).

In addition, the moles of generated gas were also calculated from GC analysis after the experiment according to Equation (2).

$$n_{gas}(mmol) = \frac{\frac{V\%_{GC}}{100} \cdot V_{gas} \cdot f_p}{V_m(T)} \qquad (2)$$

In Equation (2), V_{gas} is the volume of the gas phase (approx. 165 mL), $V\%_{GC}$ is the volume percentage of individual gas (e.g. H_2), f_p is a pressure correction factor that considers the overpressure in the photoreactor when taking the gas sample, and $V_m(T)$ is the molar gas volume at a given temperature (24.4 L mol^{-1} at 293.15 K).

2. PHBH: PRODUCTION AND BASE CHARACTERIZATION

PHBH containing *Ralstonia eutropha* Re2058/pcB113 cells were produced in a pilot stirred tank bioreactor from pork-based waste animal fat with as described previously.[1]

PHA-Extraction and precipitation

For the extraction of the *scl*-*mcl*-copolymer PHBH acetone and 2-propanol were used as suitable solvent and nonsolvent pair for extraction and precipitation of the polymer.[2] For the preparative recovery a volume to weight ratio of 10:1,5 (acetone: freeze-dried cells) was used and 3 L Solvent and 450 g cells were extracted in a 5 L round-bottom flask of a rotary evaporator in a water bath at 50 °C for 30 min. The PHA separation from cell debris was done in a centrifuge (4000 rpm, 10 min, room temperature) and the sediment was used for a second extraction step. The supernatants were collected and were concentrated using a rotary evaporator and precipitated with 4 °C cold 2-propanol in a ratio of 20:80 overnight. PHA was recovered by centrifugation at 4000 rpm for 10 min and dried under a fume hood at room temperature.

Two-stage extraction

The residual cells of two batches from extraction step 1 were pooled for a second extraction cycle. The material was extracted as described above and used with the material from the first extraction for the purification step.

Purification of the product

In order to achieve a higher purity of the PHA material, the material was dissolved again as described above and precipitated in the same way with 2-propanol. The product was dried again and used for the tests. The residual cells were dried at 50 °C. The results were analyzed regarding HHx content, and molecular weight characteristics as described previously.[2] The PHBH used in this study as starting material had an molar HHx content of 14.9 mol% an average molecular weight of 188 kDa and a polydispersity index of 2.4.

The main chemical structure of PHBH together with its optical behavior is given in **Figure S4**. Structural properties of PHBH were characterized by ^{1}H-NMR, ^{13}C-NMR, UV/VIS, and FTIR (**Figure S5**). The thermal stability of PHBH was analyzed by TGA and DSC (**Figure S6**).

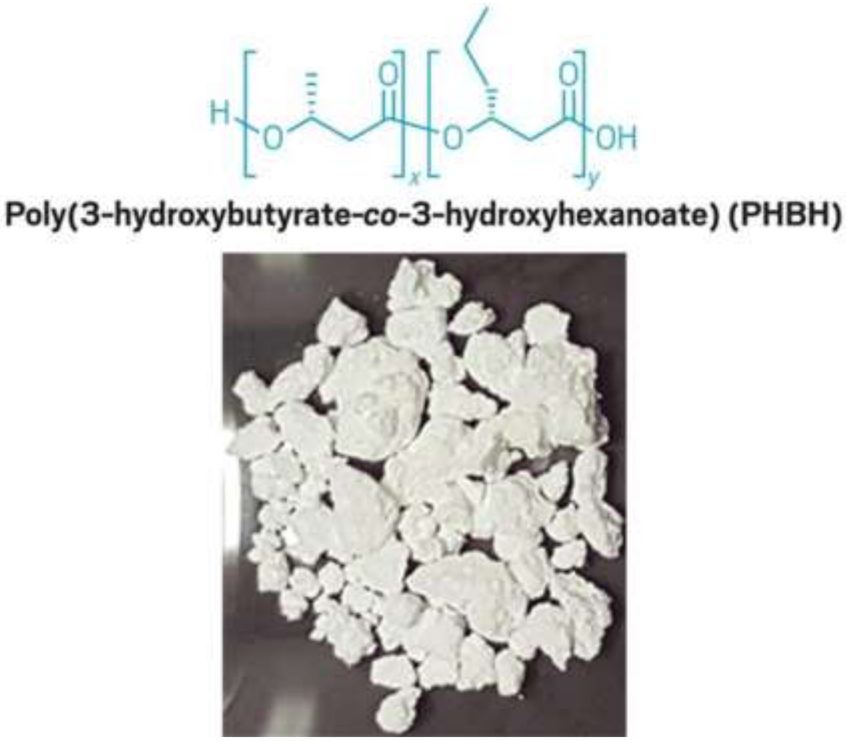

Figure S4. Chemical structure and optical behavior of PHBH.

The structural characterization shows no specific UV/Vis absorption. The FTIR spectra shows several peaks from which one at 1721 cm^{-1} is specific for the C=O group in PHBH. All peaks were identified based on spectra tables and are summarized in

Table S1. ^{1}H-NMR and ^{13}C-NMR spectra were evaluated as follows:

^{1}H-NMR (400 MHz, Acetone) δ 5.24 (q, J = 6.6 Hz, 1H), 2.80 (s, 1H), 2.60 (m, J = 5.6 Hz, 1H), 1.59 (t, J = 6.7 Hz, 1H), 1.35 (t, J = 7.4 Hz, 1H), 1.27 (d, J = 6.3 Hz, 1H), 0.92 (t, J = 7.3 Hz, 1H).

^{13}C NMR (400 MHz, Acetone) δ 206.23, 170.10 (d, J = 3.7 Hz, 1C), 71.21, 69.82 (d, J = 281.9 Hz, 1C), 41.33, 38.38 (d, J = 309.7 Hz, 1C), 20.09, 19.10, 14.26.

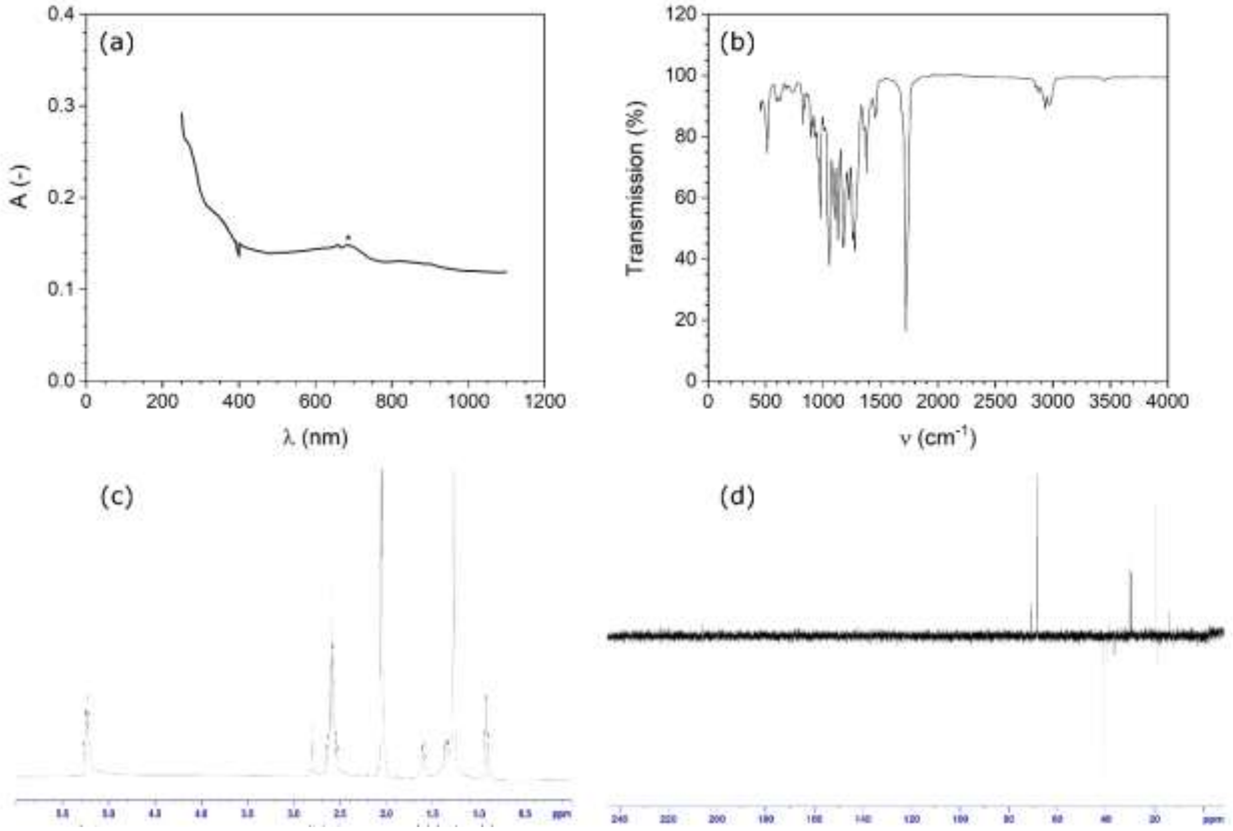

Figure S5. UV/VIS (a), FTIR (b), ^{1}H-NMR (c), and ^{13}C-NMR (d) analysis of PHBH.

Table S1. Characteristic groups of PHBH based on FTIR analysis.

**ν (cm^{-1})**	**Group**
3442	O-H
2973	CH_3
2939	CH_2
2879	C-O
2852	C-O
1721	C=O
1452	CH_2
1378	CH_3
1278	C-O-C
1261	C-O-C
1225	C-O
1177	C-O
1130	C-O
1098	C-O
1056	C-O
977	C-H
897	C-H
829	C-H
511	n.d.

The biopolymer PHBH shows a good thermal stability and decomposes above 250 °C as shown by the mass loss in TGA and the peak in DSC.

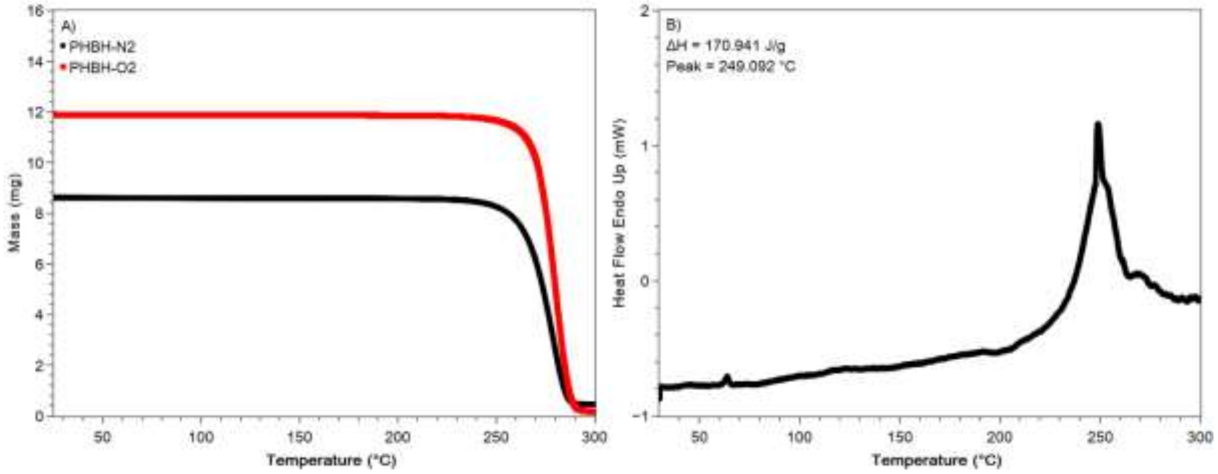

Figure S6. TGA (A) and DSC (B) analysis of PHBH.

3. Additional characterization of PHBH and PHBH/Pt1%@PC500 films

UV/Vis spectra of PHBH films on glass prepared at different PHBH concentrations and temperatures were recorded in the range of 200 – 800 nm (not shown). For the visible range (400 – 800 nm), the mean absorption values were calculated and visualized in a 2D contour plot to indicate film transparency (**Figure S7)**. Higher transparency is observed for PHBH films prepared at higher temperatures.

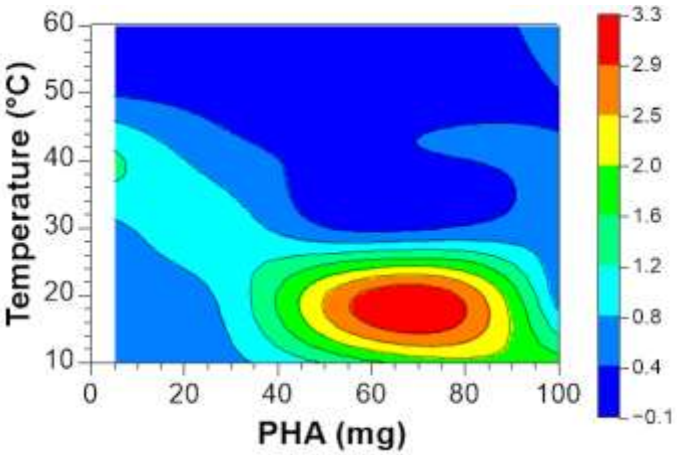

Figure S7. Mean UV/Vis absorption in the range of 400 to 800 nm for PHBH films shown in **Figure 4** in the main text.

The PHBH films are very hydrophobic (**Figure S8A**), but become more hydrophilic in the presence of the photocatalyst (**Figure S8B**).

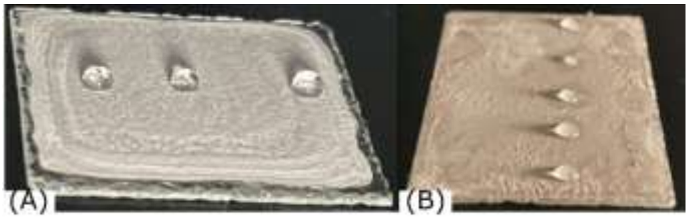

Figure S8. Water droplets on PHBH surface (left) and PHBH/Pt1%@PC500 surface (right).

To verify the presence of PHBH or PHBH/Pt1%@PC 500 on the glass substrate and to investigate the impact of the PHBH to Pt1%@PC 500 ratio as well as immobilization temperature, XRD measurements were performed. The XRD pattern are shown in **Figure S9**.

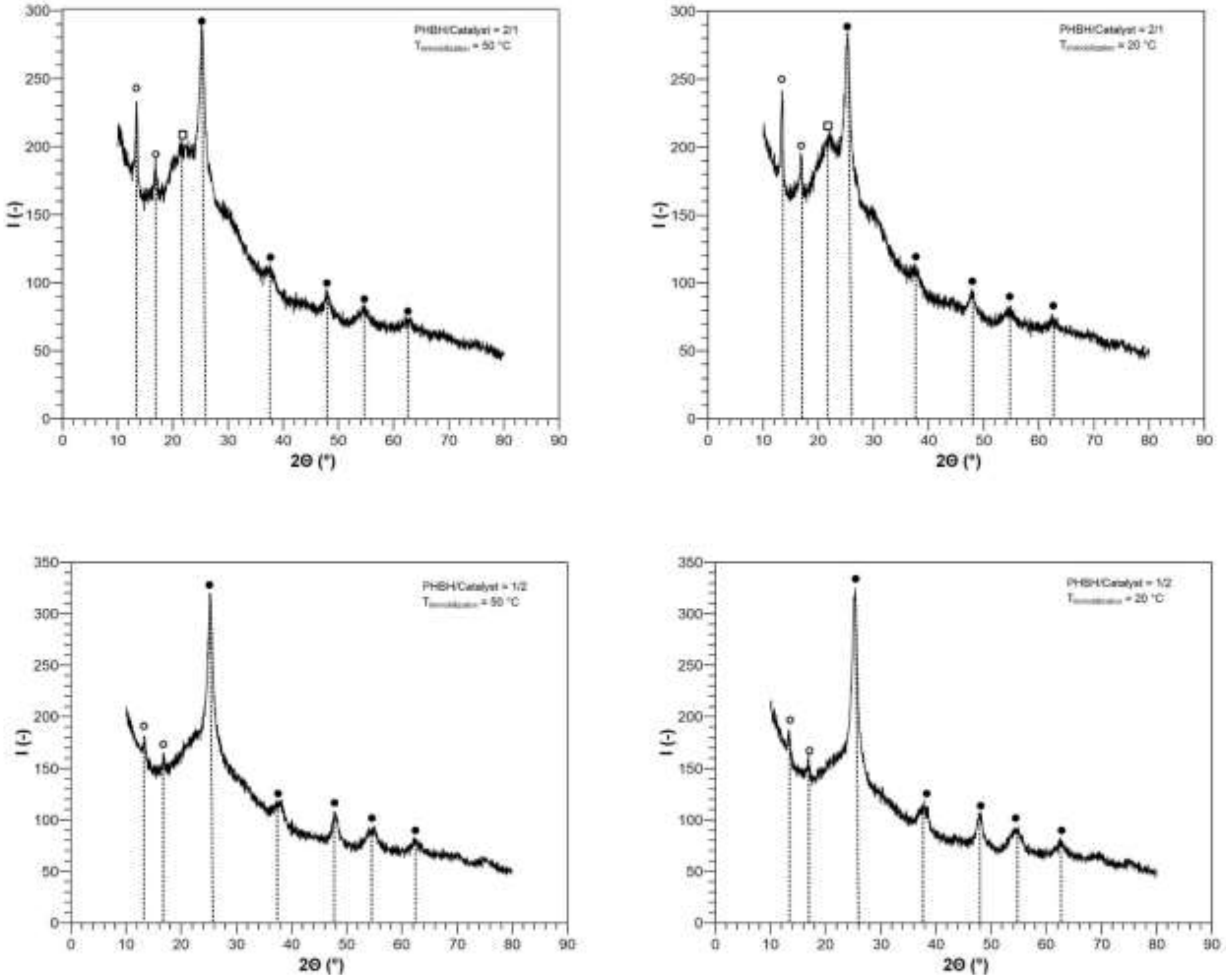

Figure S9. XRD pattern to verify the presence of Pt1%@PC 500 (closed cycles) supported on the glass substrate (open square) and to show the effect of PHBH (open cycles) as well as immobilization temperature ($m_{PHBH+Photoctalyst}$ = 14.1 mg).

The applied PC500 photocatalyst consists of mainly anatase phase and shows 2Θ diffraction peaks at 25.0, 37.9, 47.7, 54.2, 62.1, 69.8, and 75.2 that correspond to the (101), (004), (200), (105), (213), (220), and (215) planes, according to JCPDS card no. 21-1272. Depending on the immobilization conditions, especially the ratio of PHBH to photocatalyst, the intensity is slightly different. In **Figure S9**, the total mass of PHBH and photocatalyst were fixed to about 14.1 mg, and in the case of higher amount of PHBH, the two diffraction peaks of PHBH (13.5°, 16.8°) are more pronounced compared to TiO_2. When comparing the largest Peak of TiO_2 with the peaks for PHBH, the ratio is a little bit higher for 50 °C. The XRD pattern does not show a similar strong temperature effect as expected from SEM images (**Figure 7** in main text), but it shows the same tendency. There is a very broad peak at about 22° that belongs to the substrate. For all immobilizations, a glass substrate was use, which is a very amorphous material.

Biopolymer films with PHBH concentrations of 1 g L^{-1}, 3 g L^{-1}, 6 10 g L^{-1}, g L^{-1}, 50 g L^{-1}, and 100 g L^{-1} were prepared by dissolving the required amount of PHBH in 1 mL of acetone. The glass substrate (2.1 cm x 3.6 cm) was placed on a heating plate and biopolymer solution was drop coated at 50 °C. Up to a PHBH concentration of about 10 g L^{-1}, a clear transparent and homogenous film was obtained (**Figure S10**, top). At higher PHBH concentrations, the viscosity of the solution increases and acetone evaporation is more challenging leading to an inhomogeneous film. The film thickness was measured increases with PHBH concentration (**Figure S10**, down), but it was not possible to measure a meaningful value up to 5 g L^{-1}. The film thickness at higher PHBH concentrations was in the range of 100-200 μm (estimated error 10%). In the case of the PHBH/Pt1%@PC500 film, where lower PHBH concentrations are used to immobilize the particles, the film thickness is larger due to the particles. In most of the cases, the PHBH film thickness in in the range of 100 μm.

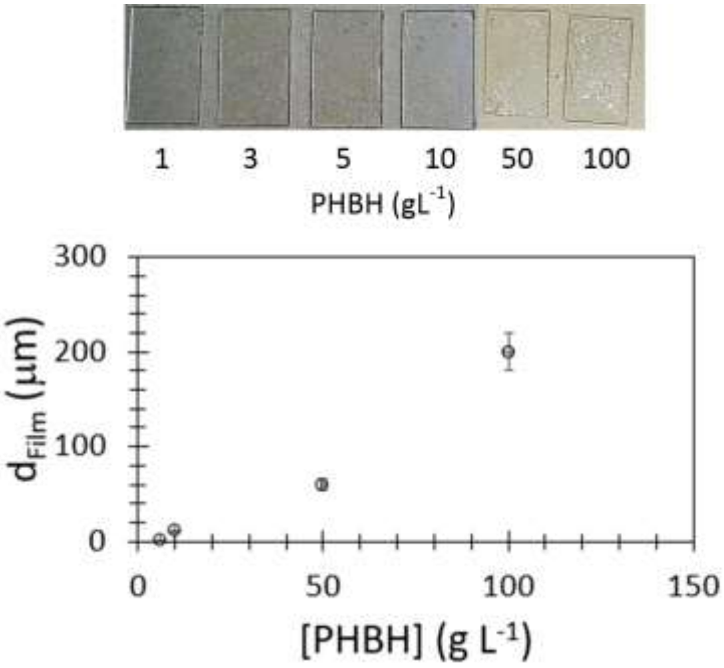

Figure S10. Prepared PHBH films at 50 °C with different PHBH concentrations (top) and measured film thickness (down).

3. Characterization of Pt1%@PC500, TEM/SEM description

The Pt1%@PC500 catalyst was prepared through reduction of the $[Pt(dba)_3]$ precursor in the presence of PC500 using hydrogen. **Figure S11** shows a homogeneous distribution of the PtNPs with a mean particle size of about 2.0 nm.

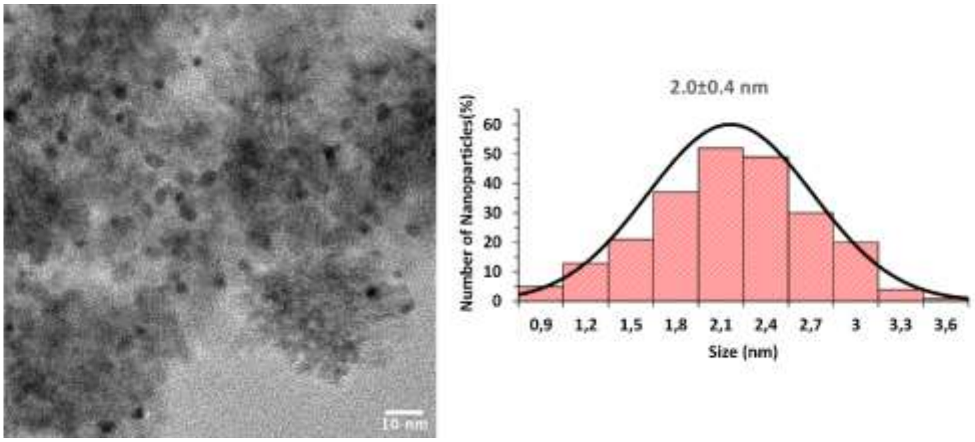

Figure S11. TEM image of Pt1%@PC500 (left) and PtNPs particle size distribution (right).

TEM: TEM/HR-TEM analyses were performed at "Servei Microscopia de la UAB" with a JEOL JEM 2010 electron microscope working at 200 kV with a resolution point of a 2.5 Ǻ. The size distributions were determined via manual analysis of enlarged micrographs by measuring ca. 200 particles on a given grid to obtain a statistical size distribution and a mean diameter.

SEM: The morphology of the prepared photocatalyst films was investigated with scanning electron microscopy (SEM) using a Hitachi SEM type SU8030 microscope operated at an acceleration voltage of 10 kV and a probe current of 15 pA.

4. Hydrogen production with PHBH/Pt1%@PC500 films

UV/Vis spectra of PC500, Pt1%@PC500, and PHBH/Pt1%@PC500 were recorded with a Lambda 365 spectrometer from Perkin Elmer (**Figure S12A**) to obtain their band gap energy from the Tauc plots (**Figure S12B**). The photocatalyst PC500 absorbs mainly UV light and the band gap energy is about 3.1 eV. The band gap energy remains the same in the presence of platinum nanoparticles as the co-catalyst and after immobilization as a PHBH/Pt1%@PC500 film. Therefore, PHBH acts only as a binder and doesn't change the characteristics of the photocatalyst.

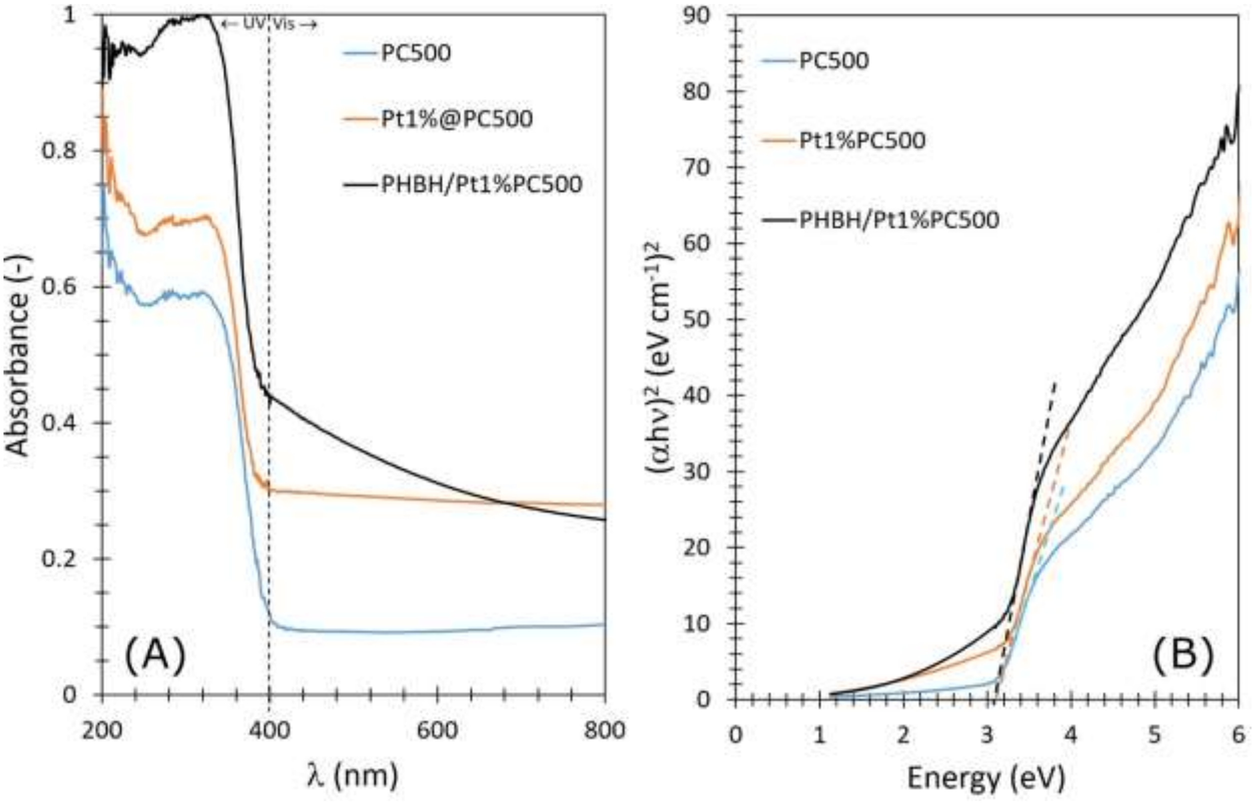

Figure S12. UV/Vis spectra (A) and determination of band gap energy (B).

When the PHBH/Pt1%PC500 film is irradiated with the UV-LED, it shows a high activity for photocatalytic hydrogen production as obvious from the production of many H_2 gas bubbles as shown in **Figure S13**. As the PHBH is hydrophobic, a larger fraction of the produced hydrogen sticks to the film surface in the case of too high PHBH concentrations.

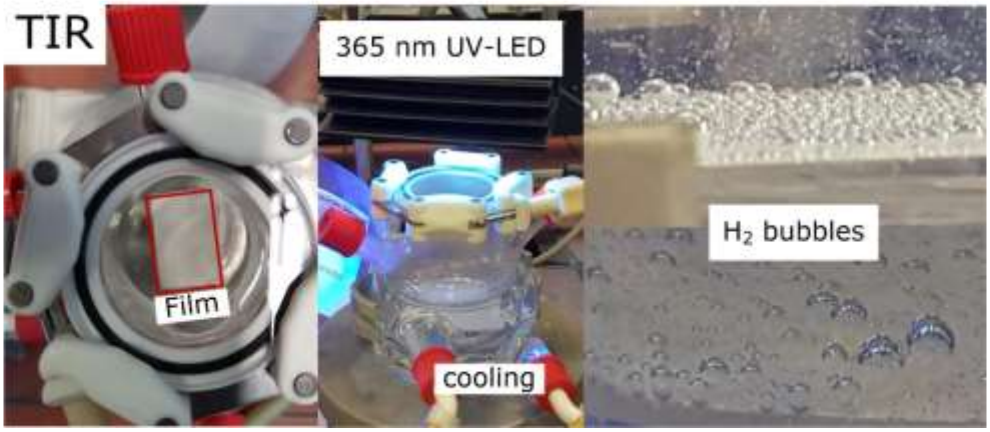

Figure S13. PHBH/Pt1%PC500 film on glass (B x H = 2.6 cm x 3.6 cm) in the top irradiation reactor (left), during irradiation with the UV-LED (middle), and produced hydrogen bubbles (right).

PHBH/Pt1%PC500 films were prepared at 20 °C and 50 °C (**Figure S14**) to study the effect of PHBH onto the film formation. At 20 °C, evaporation of acetone is slow and PHBH covers the Pt1%@PC500 particles. At 50 °C, evaporation of acetone is fast and more Pt1%@PC500 particles are at the surface.

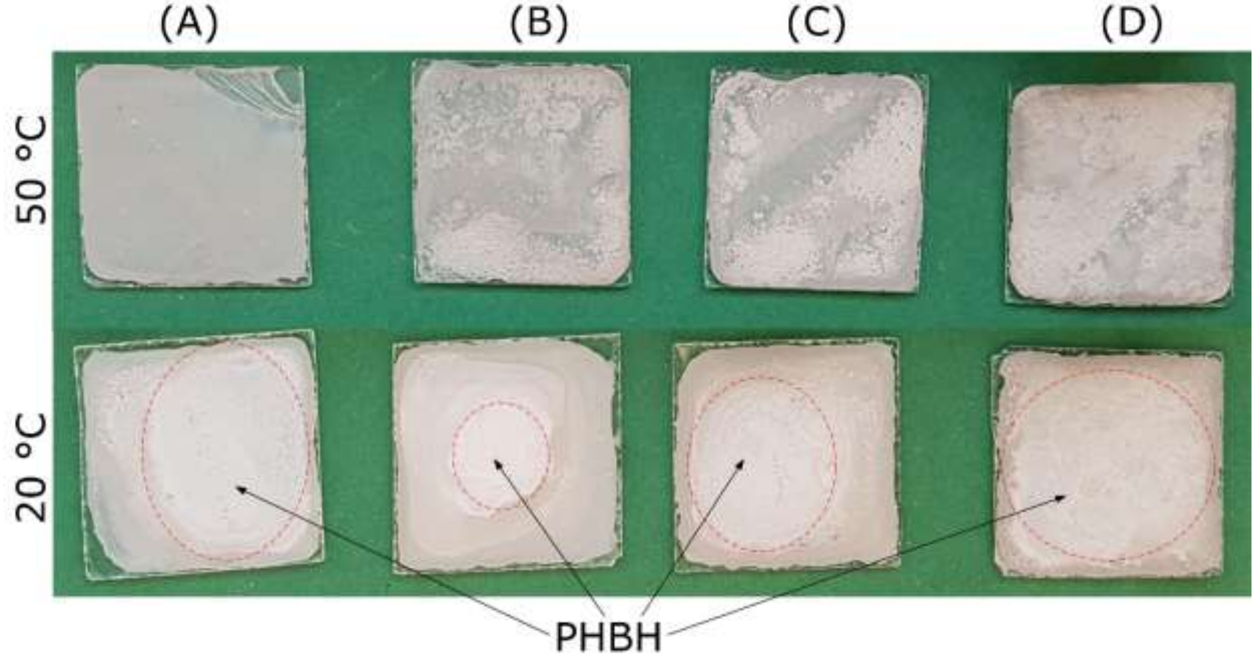

Figure S14. PHBH/Pt1%PC500 films on glass (B x H = 2 cm x 2 cm) prepared at 20 °C and 50 °C with different Pt1%@PC500 amounts (m_{PHBH} = 4.3 mg, L_{PHBH} = 1.1 mg cm^{-2}, $m_{Pt1\%@PC500}$ = 2.2 mg (A), 4.3 mg (B), 6.4 mg (C), and 8.6 mg (D).

When the PHBH layer is formed on top, it lowers the activity. **Figure S15** shows the initial photocatalytic activity of hydrogen production for samples prepared at 20 °C and 50 °C. The sample prepared at 50 °C shows a higher activity as the surface is less covered by the PHBH. Further, samples prepared at 50 °C show a better stability. When the films were prepared at 20 °C, film detachment from the glass substrate was observed during hydrogen production.

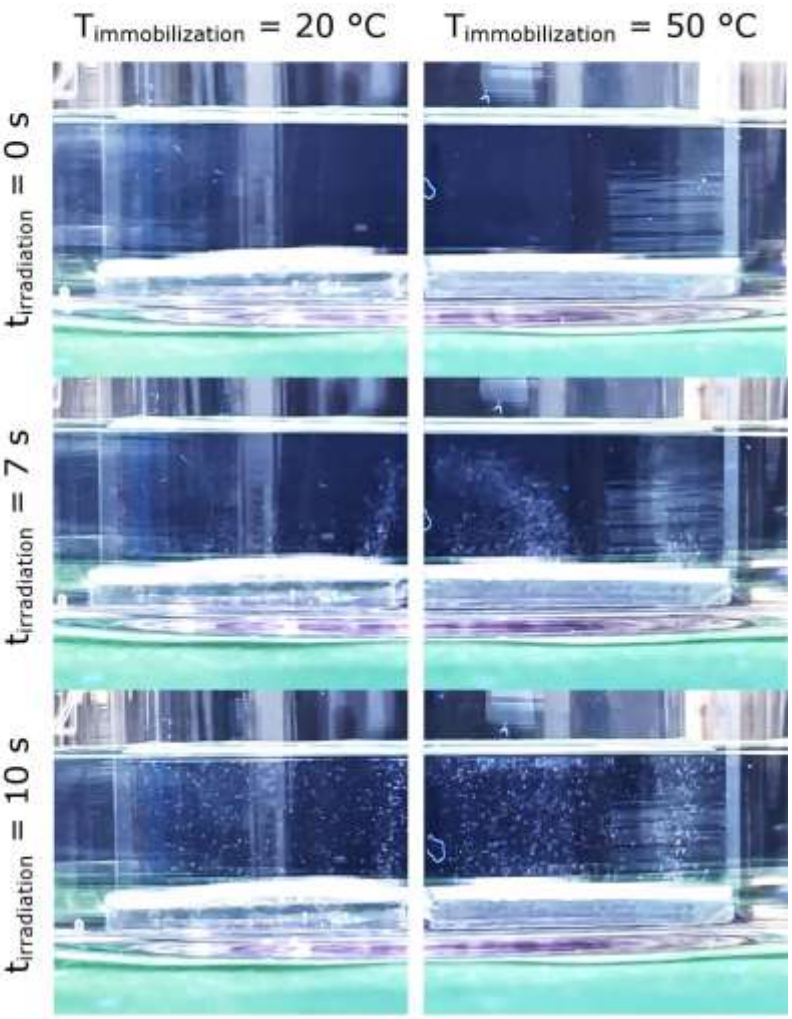

Figure S15. Initial photocatalytic activity of samples prepared at two different temperatures.

A PHBH/Pt1%@PC500 film was prepared on glass substrate (rough surface, A = 2.6 cm x 3.6 cm) at 50 °C using 5.7 mg PHBH, 6.1 mg Pt1%@PC500, and 7 mL acetone as the casting solution. The hydrogen production was investigated in the top-irradiation reactor (conditions: 20 mL aqueous solution containing 10 vol% EtOH, T = 20 °C). **Figure S16** shows the cumulative hydrogen production (a), the area-based hydrogen production rate (b), the mass loss (c), and the optical behavior of the film after each run. The film shows a quite stable photocatalytic performance with some observed mass losses, which are partially indicated by red cycles. It is obvious that the base film structure is kept through the six runs.

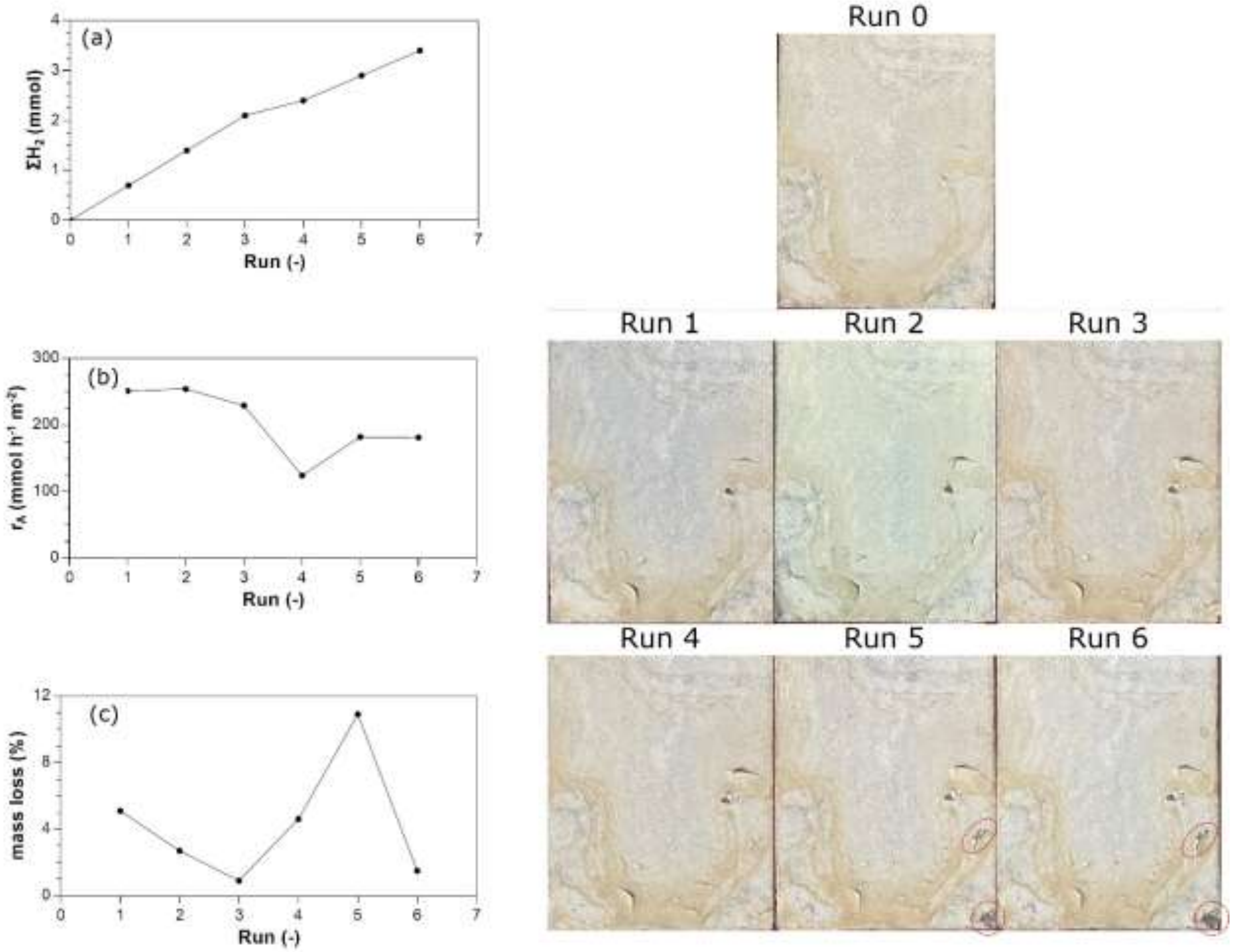

Figure S16. Recycling experiment with PHBH/Pt1%PC500 film. Cumulative hydrogen production (a), area-based hydrogen production rate (b), relative mass loss (c), and photos of the film before use (Run 0) as well as after each run (each run was 3h). Exemplarily mass losses are indicated by red cycles.

In order to get a better insight into the long-term stability of the film, a test was carried out over 98 hours. As the productivity of the film was high, which led to an increase in pressure, the test could not be carried out in the reactor with top irradiation. Instead, a photoreactor was used that can be operated at up to 1.5 bar overpressure (see **Figure S3**). As the total pressure after the 98-

hour test would exceed the maximum permissible pressure, the test was divided into three runs. The first run was 24 hours, the second one was 50 hours, and the last one was again 24 hours. The overpressure (not shown) for the first, second, and third run was 1.0 bar, 0.8 bar, and 0.4 bar, respectively (volume of the gas phase approx. 165 mL; T = 20 °C). As described above, the ideal gas law can be used to calculate the amount of produced gas in mmol from the pressure increase. As shown in **Figure S17**, about 6 mmol gas was produced in run 1, about 5 mmol in run 2, and about 2 mmol in run 3.

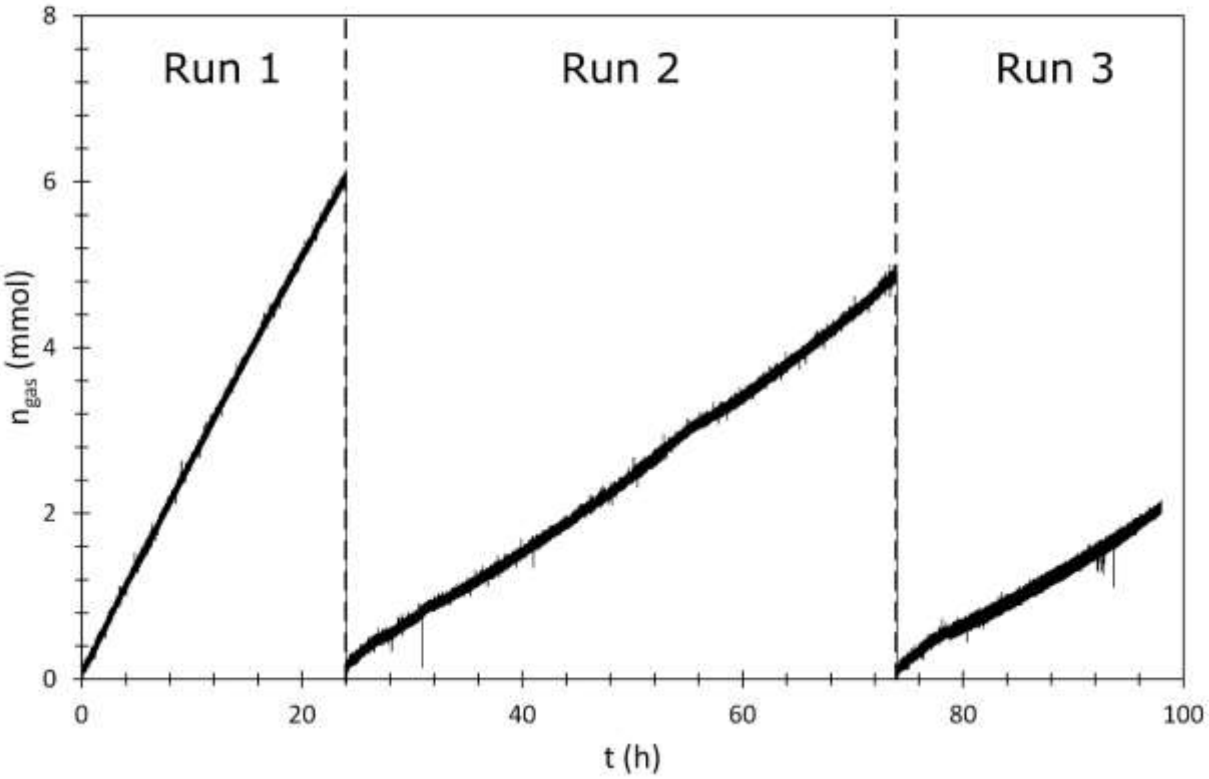

Figure S17. Long-term hydrogen evolution with a PHBH/Pt1%@PC500 film (V_L = 35 mL; 20 vol% EtOH; V_{gas} = 165 mL; A_{film} = 1.5 cm x 3.5 cm; L_{PHBH} = 0.5 mg cm^{-2}; PHBH/Pt1%@PC500 = 1/1; 365 nm UV-LED; T = 20 °C).

After each run, a sample of the gas phase was taken and analyzed by GC to determine the composition of the gas phase. In addition to hydrogen (H_2), carbon dioxide (CO_2), carbon monoxide (CO), methane (CH_4), ethane (C_2H_6), and ethylene (C_2H_4) were also produced. However, with a mole fraction of about 90%, hydrogen is the main product.

Table S2. Composition of gas phase after each run obtained from GC measurement and produced amounts of gas (mean values from two GC injections; Error: ± 10%).

**Run #**	**H_2 (vol%)**	**CO_2 (vol%)**	**CH_4 (vol%)**	**C_2H_6 (vol%)**	**C_2H_4 (vol%)**	**CO (vol%)**
1	36.9	2.0	2.1	0.2	0.02	0.005
2	31.4	1.0	1.1	0.09	0.05	0.006
3	19.0	0.9	0.8	0.08	0.09	0.006
**Run #**	**H_2 (mmol)**	**CO_2 (mmol)**	**CH_4 (mmol)**	**C_2H_6 (mmol)**	**C_2H_4 (mmol)**	**CO (mmol)**
1	4.991	0.270	0.284	0.028	0.003	0.0007
2	3.858	0.120	0.135	0.011	0.007	0.0008
3	1.832	0.084	0.074	0.008	0.009	0.0005

After 98 hours irradiation, the film was dried at 65 °C and weighted. No mass loss was detected. The base structure of the film was retained, but the film appeared darker at the edges (**Figure S18**). A change in the film morphology due to handling between the run (e.g., vacuum treatment) and gas evolution during the runs is assumed. Also the film slighted detached at the edges.

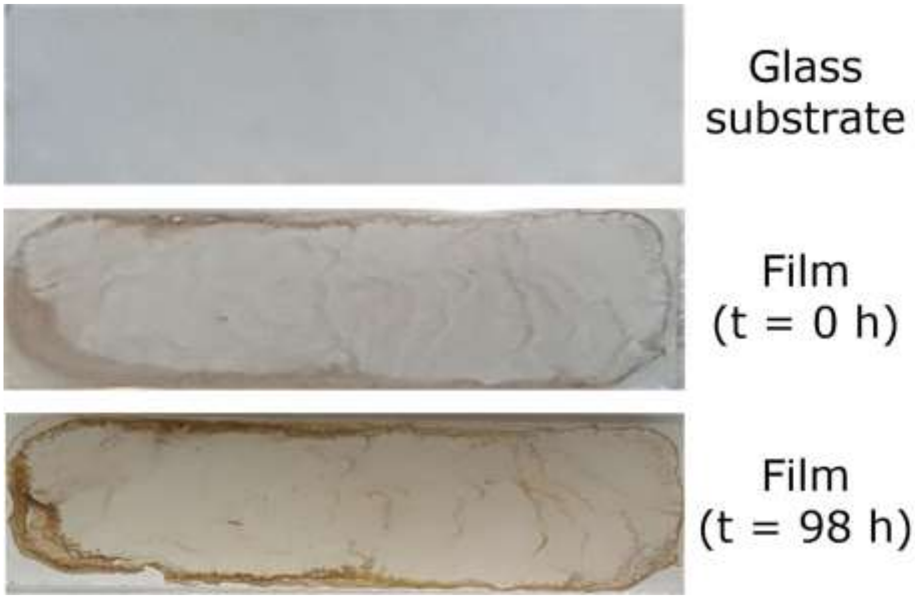

Figure S18. Photos of glass substrate and film before and after irradiation.

References

(1) Gutschmann, B.; Maldonado Simões, M.; Schiewe, T.; Schröter, E. S.; Münzberg, M.; Neubauer, P.; Bockisch, A.; Riedel, S. L. Continuous Feeding Strategy for Polyhydroxyalkanoate Production from Solid Waste Animal Fat at Laboratory- and Pilot-scale. *Microb. Biotechnol.* **2023**, *16* (2), 295–306. https://doi.org/10.1111/1751-7915.14104.

(2) Bartels, M.; Gutschmann, B.; Widmer, T.; Grimm, T.; Neubauer, P.; Riedel, S. L. Recovery of the PHA Copolymer P(HB-Co-HHx) With Non-Halogenated Solvents: Influences on Molecular Weight and HHx-Content. *Front. Bioeng. Biotechnol.* **2020**, *8*, 944. https://doi.org/10.3389/fbioe.2020.00944.
